# Role of reactive oxygen species in myelodysplastic syndromes

**DOI:** 10.1186/s11658-024-00570-0

**Published:** 2024-04-14

**Authors:** Qiangan Jing, Chaoting Zhou, Junyu Zhang, Ping Zhang, Yunyi Wu, Junyu Zhou, Xiangmin Tong, Yanchun Li, Jing Du, Ying Wang

**Affiliations:** 1 Laboratory Medicine Center, Department of Clinical Laboratory, Zhejiang Provincial People’s Hospital (Affiliated People’s Hospital), Hangzhou Medical College, Hangzhou, 310014 Zhejiang China; 2 HEALTH BioMed Research & Development Center, Health BioMed Co., Ltd, Ningbo, 315803 Zhejiang China; 3 Department of Hematology, Lishui Central Hospital, Lishui, 323000 Zhejiang China; 4 Department of Central Laboratory, Affiliated Hangzhou First People’s Hospital, School of Medicine, Westlake University, Hangzhou, 310006 Zhejiang China

**Keywords:** Reactive oxygen species, Oxidative stress, Myelodysplastic syndromes, Hematological niche

## Abstract

Reactive oxygen species (ROS) serve as typical metabolic byproducts of aerobic life and play a pivotal role in redox reactions and signal transduction pathways. Contingent upon their concentration, ROS production not only initiates or stimulates tumorigenesis but also causes oxidative stress (OS) and triggers cellular apoptosis. Mounting literature supports the view that ROS are closely interwoven with the pathogenesis of a cluster of diseases, particularly those involving cell proliferation and differentiation, such as myelodysplastic syndromes (MDS) and chronic/acute myeloid leukemia (CML/AML). OS caused by excessive ROS at physiological levels is likely to affect the functions of hematopoietic stem cells, such as cell growth and self-renewal, which may contribute to defective hematopoiesis. We review herein the eminent role of ROS in the hematological niche and their profound influence on the progress of MDS. We also highlight that targeting ROS is a practical and reliable tactic for MDS therapy.

**Graphical Abstract:**

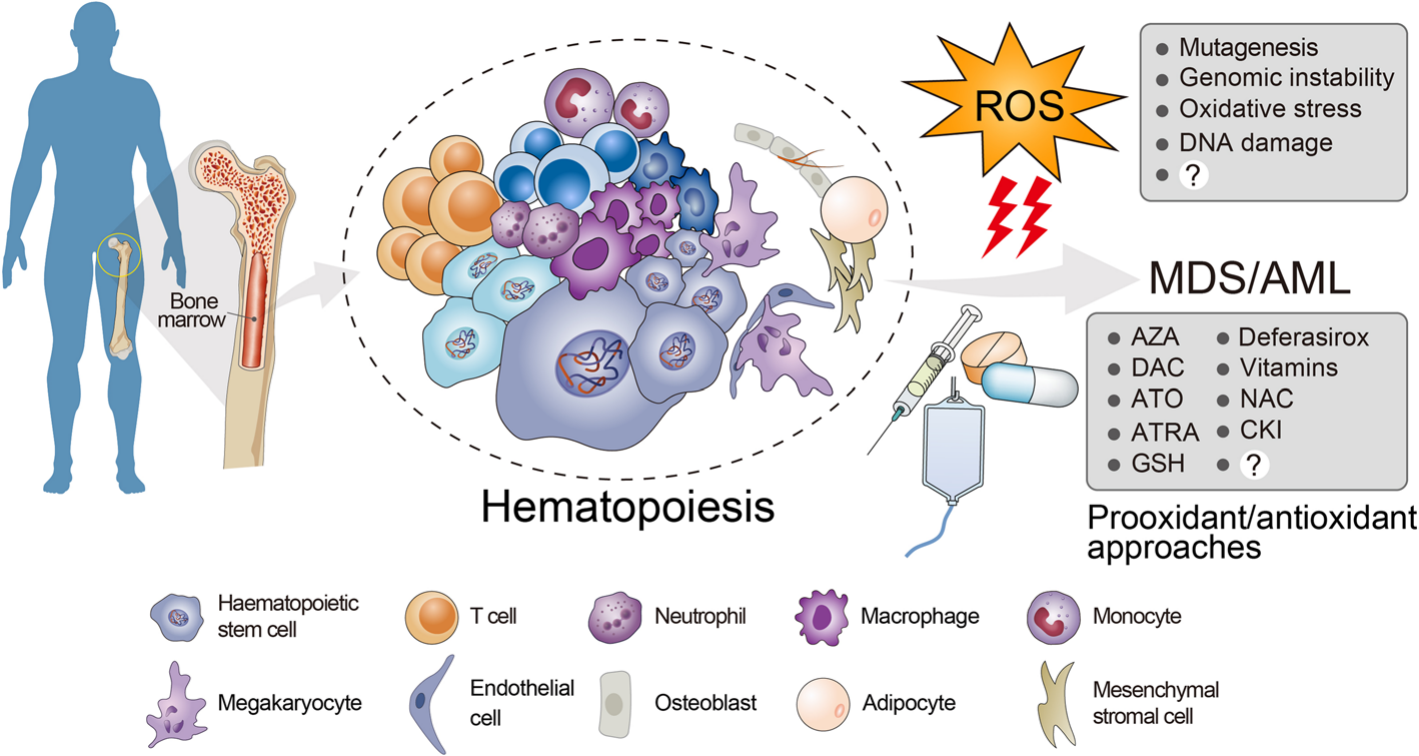

## Introduction

Over the past few decades, despite great advancements in therapy, cancer remains a key challenge to human health and a leading cause of death globally [1]. Triggering apoptotic signaling pathways using anticancer drugs to induce apoptosis is one of the principal strategies for cancer treatment [2, 3]. However, the complicated pathogenesis and acquired or intrinsic resistance of several cancers make it difficult to kill cancer cells effectively using therapeutic avenues, such as chemotherapy and radiotherapy [4]. Therefore, insights into the endogenous or exogenous factors influencing the etiology are important for eliminating cancer cells. Reactive oxygen species (ROS) are byproducts of cell growth under aerobic conditions and are mainly derived from mitochondrial metabolism [5]. Specifically, ROS encompass a group of derivatives of molecular oxygen [e.g., superoxide anion radical (O_2_^⋅−^), hydroxyl radicals (OH^⋅^), hydrogen peroxide (H_2_O_2_), and singlet oxygen (^1^O_2_)], which are formed by redox reactions or electron transfer in the mitochondrial electron transport chain (ETC) [6]. One of the prominent hallmarks of cancer cells is their high metabolic rate and uncontrolled proliferation [7]; therefore, they maintain higher ROS production and exhibit more aberrant redox homeostasis than noncancerous cells [8]. Several studies have emphasized that many transcription factors involved in the regulation of redox homeostasis are activated by ROS [9]. In some cancers, low or moderate levels of ROS could promote cell proliferation, differentiation, metastasis, and even chemoresistance, protecting cells from cytotoxic ROS by acting as signaling molecules to activate antioxidant systems in response to stress [5, 10–12]. ROS, which are important signaling molecules, are often closely involved in the pathogenesis of numerous diseases and influence tumorigenesis, such as myelodysplastic syndromes (MDS) and chronic/acute myeloid leukemia (CML/AML) [13–17].

In all healthy cells, the regulation of redox homeostasis is essential for cellular maintenance, proper execution, and survival. However, numerous pathological states are characterized by an aberrant redox state in which the generation and elimination of ROS are imbalanced, leading to oxidative stress (OS) [18]. OS is closely associated with many pathological conditions, such as aging [19], Parkinson’s disease [20], Alzheimer disease [21, 22], rheumatoid arthritis [23], cardiovascular diseases [24, 25], neurodegenerative diseases [26, 27], diabetes [28], and cancer [29]. Compelling evidence has highlighted that chronic OS affects the progression of several hematological malignancies, including MDS and leukemia [30–32]. In this context, ROS are significant factors in tumor formation and the response to antineoplastic therapy, and the role of ROS in inhibiting or promoting malignant tumor onset may be determined by OS. This review aims to investigate the role of ROS in MDS and discuss whether ROS is an attractive therapeutic target for MDS treatment.

## Formation of ROS and OS

**Fig. 1:**
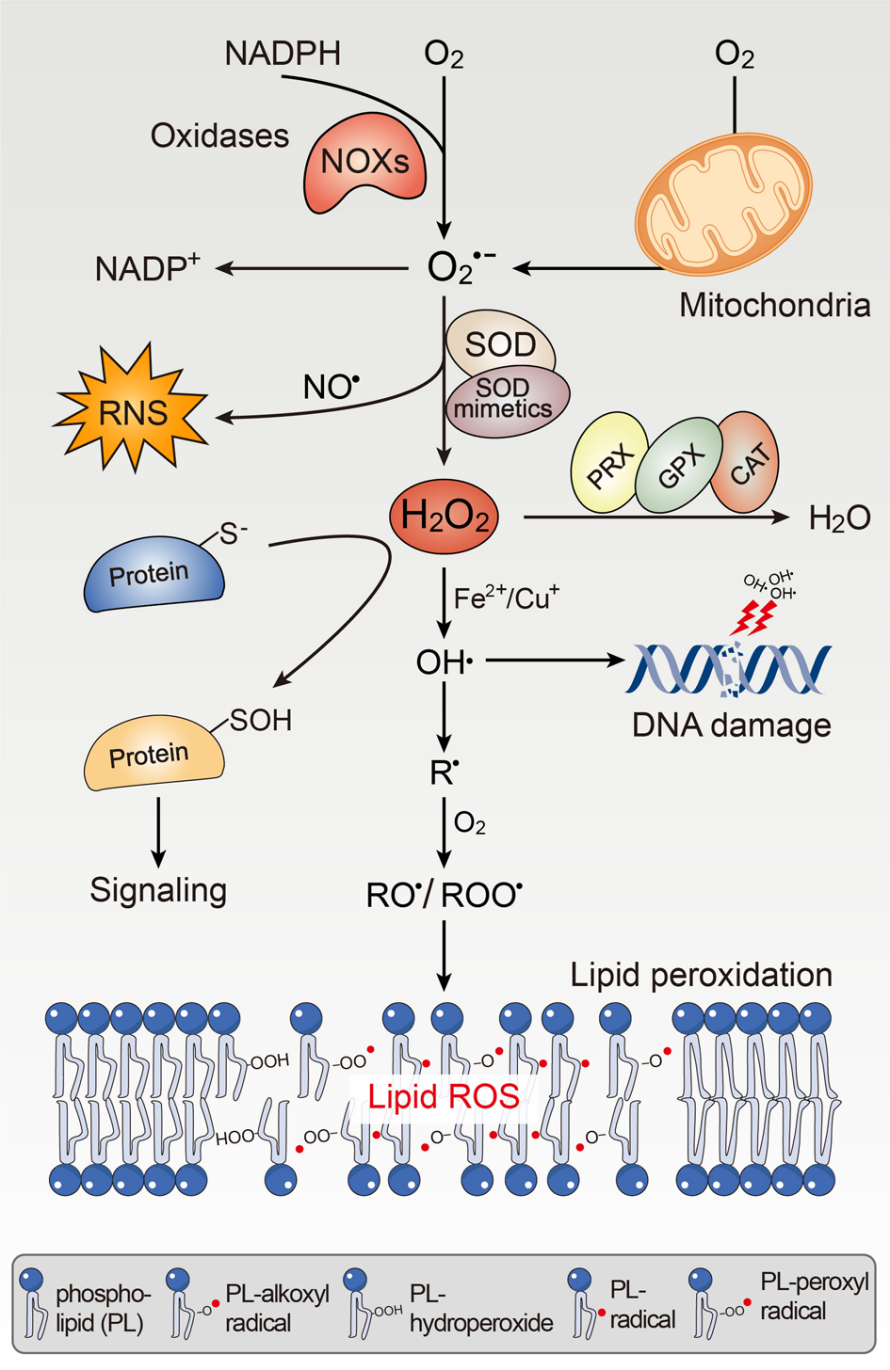
Basics of ROS. The formation of intracellular O_2_^⋅−^ could be deemed as a result of the activity of NOXs, or oxidative phosphorylation in mitochondria. Superoxide molecule as a reductant or an oxidant lies at the hub of a series of redox reactions. Mostly, superoxide radicals are catalyzed to H_2_O_2_ by superoxide dismutases, including cytosolic SOD1, mitochondrial SOD2, and extracellular SOD3. Alternatively, superoxide reacts with NO^⋅^ to form strong oxidative ONOO^−^, which can mediate oxidative modification of protein residues and induce RNS production. Physiological levels of H_2_O_2_ are strictly regulated by multiple mechanisms, such as acting with PRX, GPX, and CAT to form H_2_O, while H_2_O_2_ is also able to oxidation cysteine residues on proteins for signaling transduction. If, however, excessive H_2_O_2_ is not controlled, it will be decomposed into OH^⋅^ in the presence of metal cations (e.g., Fe^2+^ and Cu^+^). OH^⋅^ can react with DNA and irreversibly damage DNA base units and also reacts with RH, forming R^⋅^. R^⋅^ further reacts with O_2_, building up RO^⋅^ or ROO^⋅^, which can cause lipid peroxidation by a series of reaction steps and ultimately subvert membrane stability and permeabilization

**Fig. 2:**
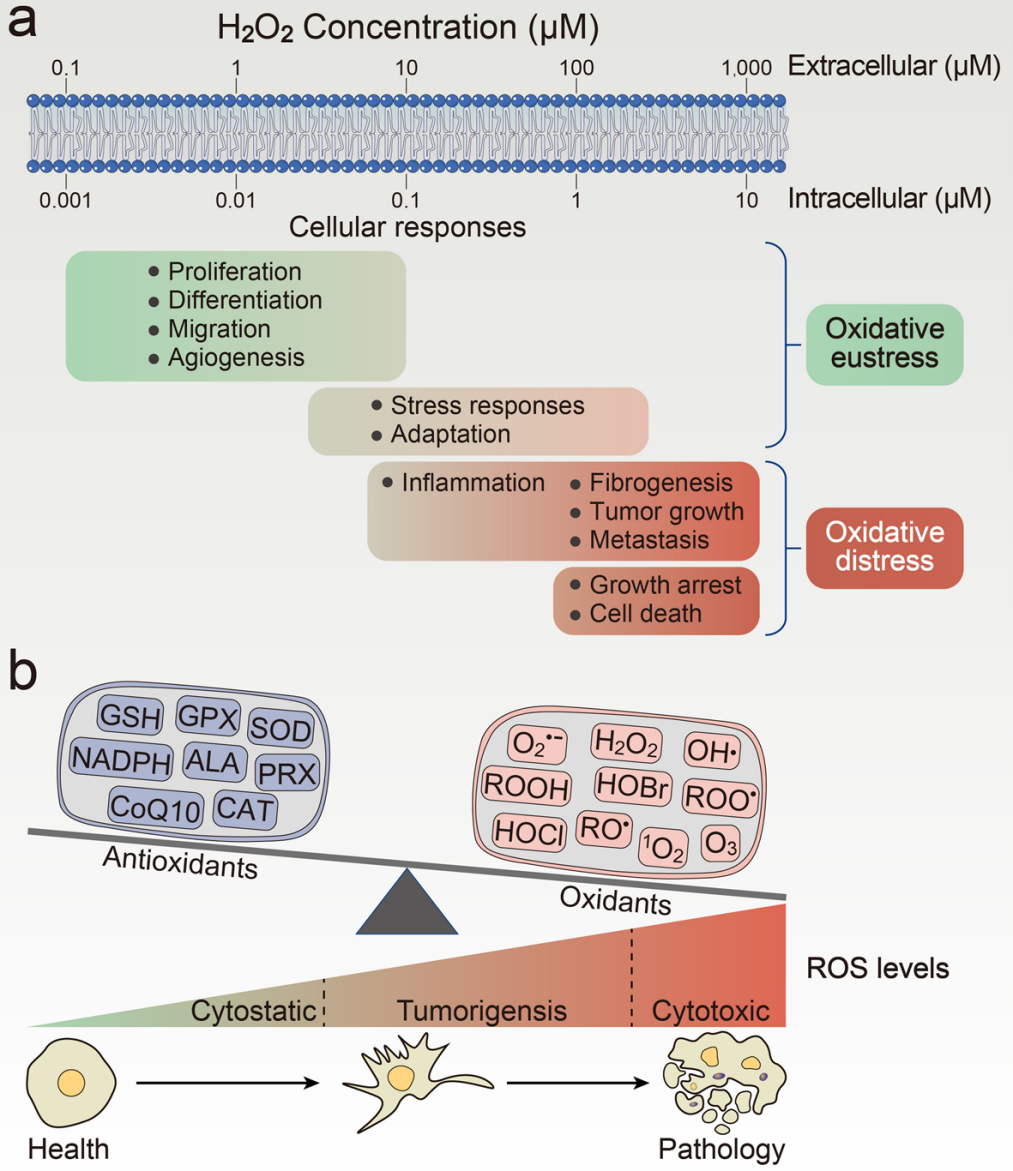
Intracellular concentration of H_2_O_2_, ROS levels, and OS. **a** Estimated ranges of H_2_O_2_ concentration concerning OS cellular responses. The intracellular physiological range of H_2_O_2_ boasts a wide span from 1 to 100 nM, and cellular proliferation, differentiation, migration, and angiogenesis rely heavily on that appropriate range. High concentrations of H_2_O_2_ trigger cellular adaptive stress responses. Even higher levels result in inflammatory responses, growth retardation, tumor growth, metastasis, and cell death through different mechanisms. Green and orange coloring stands for principally eustress and distress responses, respectively. It is estimated that a 100-fold concentration gradient, which varies with cell type, the location inside cells, and the activity of enzymatic sinks, gives a rough orientation from extracellular to intracellular [18, 279]. **b** Imbalance between oxidants and antioxidants causes OS and influences tumorigenesis. Excessive ROS generation leads to prooxidative/antioxidative imbalance and OS, which could be detrimental and result in cellular dysfunction or cell death. For tumor cells, a higher generation of ROS and an elevated redox state are crucial for tumorigenesis. In addition, tumor cells are able to increase the antioxidant levels to alleviate the cytotoxic effect of ROS and counteract OS-induced cell death

Numerous physiological processes are accompanied by the formation of ROS and reactive nitrogen species (RNS), which are unavoidable consequences of cellular metabolism. ROS can be defined as nonradicals and free radicals (with one or more unpaired electrons) derived from diatomic oxygen. Highly reactive superoxide radicals (O_2_^⋅−^) derived from the monovalent reduction of oxygen are at the heart of a range of potential chemical reactions [9], as well as the first step of ROS production (Fig. 1); For example, superoxide radicals can react with nitric oxide and mediate RNS production. Commonly, rapid superoxide reactions with superoxide dismutases (SODs) yield the versatile signaling molecule hydrogen peroxide (H_2_O_2_). H_2_O_2_, a membrane-permeable and moderately prooxidant molecule, is a key agent in redox signaling, and its production is controlled by metabolic cues or numerous stress factors, including growth factors, chemokines, and physical stressors [33]. The elimination of H_2_O_2_ is implemented by peroxiredoxins (PRX), glutathione peroxidase (GPX), and catalase (CAT) in the thioredoxin (Trx) and glutathione (GSH) systems [34]. In the low nanomolar range (intracellular concentrations below 100 nM), H_2_O_2_ mediates the reversible oxidation of cysteine residues via specific protein targets and participates in the regulation of metabolic activity in response to external stress [9, 35, 36]. However, a supraphysiological concentration of H_2_O_2_ (above 100 nM) can irreversibly modify and cause permanent impairment of DNA, proteins, or biomolecules [36, 37], eventually leading to cell growth arrest or even senescence and death, a condition known as OS (Fig. 2a), which is why cells have evolved professional defense mechanisms to control and scavenge the accumulation of H_2_O_2_ and often maintain it at low or nontoxic threshold concentrations. Furthermore, the Fenton reaction, which mostly involves the decomposition of excess H_2_O_2_ catalyzed by redox metals (e.g., Fe^2+^ and Cu^+^), is the primary source of deleterious hydroxyl radicals (OH^⋅^) [38]. The accumulation of hydroxyl radicals can damage DNA, resulting in genomic instability, which is significant in the etiology and pathogenesis of multiple tumors as well as in protein structure and cellular membrane devastation by initiating lipid peroxidation [39]. Thus, maintaining the homeostasis of free labile ferrous iron and cuprous ions is critical for cells to take precautions against the formation of hydroxyl radicals. The more perturbed the homeostasis of transition metal cations, the more cellular impairment is induced by toxic hydroxyl radicals or metal ions.

OS can be considered to be a disorder in which ROS generation and elimination are unbalanced, being inextricably linked to the pathology of many diseases [40], including various carcinomas. Cells have complicated biochemical and epigenetic mechanisms that maintain a relatively steady condition between prooxidative and antioxidative systems, and their disruption can result in physiological and pathological implications. Through antioxidant defense mechanisms involving both enzymatic and nonenzymatic antioxidants [41, 42], cells maintain low or moderate ROS levels under normal physiological conditions, enabling cell growth and development. Further, in the functionality of cells, ROS serve as signaling agents that can drive gene and protein expression also but are protumorigenic [43, 44]. As overproliferation and aberrant metabolism of tumors are commonly accompanied by high ROS generation, tumor cells adapt to the oxidative burden and maintain a high antioxidant status to avoid the cytotoxicity of high ROS levels [29]. However, excessive ROS reaching an uncontrolled or unscavenging status results in senescence and cellular death. Therefore, antioxidative defenses are of great significance in both signal transduction and counteracting ROS, which can maximally protect biomolecules against oxidative damage. It is noteworthy that many cellular physiological processes, such as proliferation and differentiation, cell growth, inflammation, and host defense, are subject to high ROS levels to a large extent, and those processes can be destroyed when the balance between uncontrolled ROS and antioxidants is affected (Fig. 2b).

Typically, cells contain a spectrum of endogenous antioxidant enzymes, such as GSH, GPX, SOD, CAT, PRX, and Trx, which can directly scavenge dangerous ROS (hydroxyl radicals, peroxyl radicals, superoxides, and lipid peroxides) and maintain intracellular redox homeostasis [45, 46]. GSH, a tripeptide composed of glutamate, glycine, and cysteine, is the most abundant intracellular antioxidant that participates in antioxidant defense, subdues ROS to homeostatic levels, and maintains the essential thiol status of proteins [47]. Two forms of GSH are possible: oxidized glutathione (GSSG), which is generated by GSH and interacts with H_2_O_2_; meanwhile, GSSG can be biotransformed into the reduced form of GSH in the presence of reductase catalyzed by nicotinamide adenine dinucleotide phosphate (NADPH) as an electron donor. Significant changes in the intracellular ratio of GSH to GSSG can be regarded as indicators of oxidative damage [48]. GSH levels play a paramount role in malignant tumor development, which is typically linked to proliferative responses and influences cell cycle progression, metastatic invasion, and resistance to chemotherapy [48–50]. In addition, clinical survival outcomes of patients with certain diseases are closely associated with GSH levels. The majority of the intracellular GSH content of some neoplastic cells is commonly regulated by GSH-related enzymes, and increased GSH levels are closely related to the activities of γ-glutamylcysteine ligase and γ-glutamyl-transpeptidase, as well as high expression of GSH-transporting export pumps.

As elevated GSH levels are capable of boosting antioxidative ability and resistance to OS, previous studies have observed that GSH levels tend to be elevated in a variety of malignant tumors, including pancreatic adenocarcinoma [51] and liver [52], ovarian [53], breast [54], and lung cancers [55]. In contrast, studies have observed reduced intracellular GSH content and high ROS levels in bone marrow cells from patients with MDS [56], indicating that these cells are under OS. These results suggest that the depletion of glutathione or inhibition of GSH-related enzymes and enhanced cell OS may be effective methods for MDS treatment. Rasool et al. [57] analyzed 50 patients with leukemia, including those with ALL and AML, and 20 healthy controls to explore various circulating biomarkers (OS markers, electrolytes, and vitamin E). They showed that enzymatic and nonenzymatic antioxidant levels (GSH, SOD, CAT, GPX, and vitamin E), platelets, and electrolytes (Ca and Mg) were decreased when compared with controls, whereas malondialdehyde levels, which can reflect OS, were significantly enhanced in the disease subtypes of leukemia. These results indicate that the pathological state in patients with MDS and those with leukemia was inextricably associated with OS.

## Source of ROS generation in hematopoietic cells

### Mitochondria and mitochondrial ETC

**Fig. 3:**
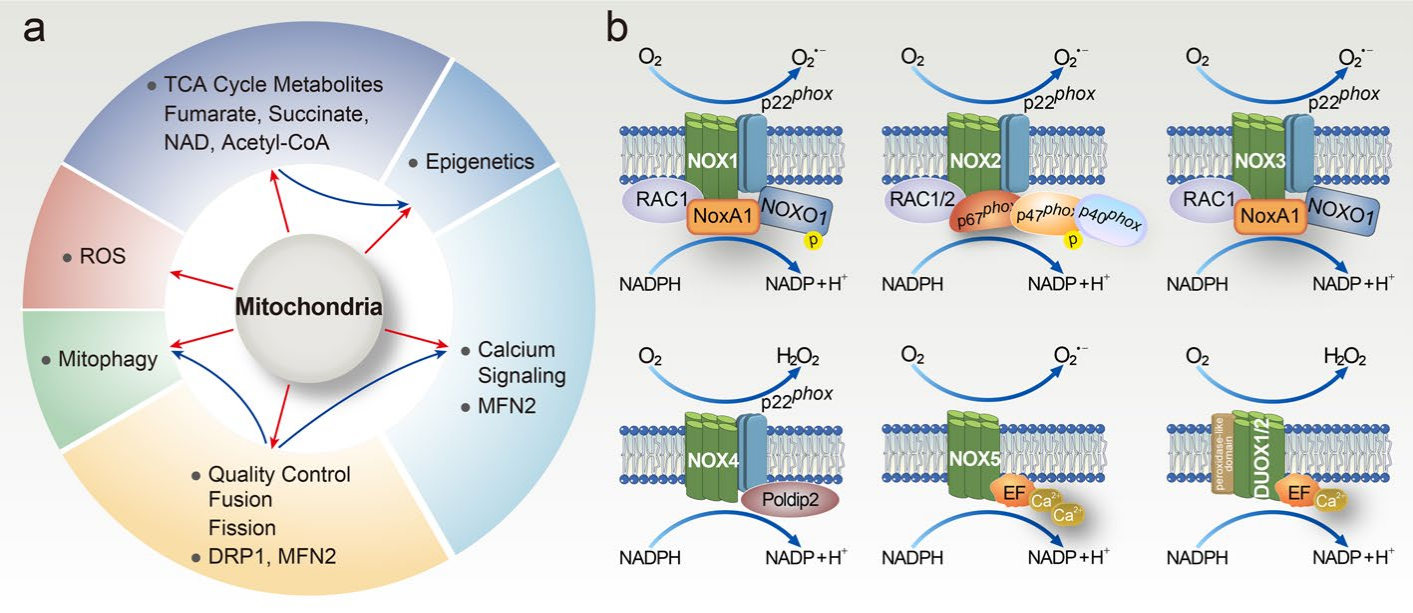
**a** Multiple mitochondrial processes regulate HSCs. HSCs are exceedingly sensitive to ROS (mainly generated by mitochondrial metabolism) levels, which can directly influence their differentiation and commitment. Excessive ROS levels invariably cause HSC pool exhaustion. Metabolites (e.g., fumarate, succinate, NAD, and acetyl-CoA), produced by mitochondria through OXPHOS and the TCA cycle, could impact the epigenetic landscape. For example, fatty acid oxidation in HSCs is required to support acetyl-CoA production. Meanwhile, the mitochondrial dynamic regulatory protein Mfn2 inhibits NFAT activity by a negative effect on intracellular calcium ions, thereby maintaining HSCs. Heightened calcium signaling prompts mitochondrial activity and participates in HSC division. In addition, mitochondrial dynamics and mitophagy are an integral part of HSC maintenance. Specifically, mitochondrial dynamics (e.g., fusion, fission, and motility) together determine mitochondrial morphology and are conducive to mitochondrial quality control and cellular stress response, while mitophagy can sweep away impaired mitochondria and contribute to the normal function of HSCs. Red arrows represent mitochondrial-related processes, while blue arrows stand for secondary effects. **b** Assembly of NOX isoforms. The NOX2 complex is composed of cytosolic subunits (p47^phox^, p40^phox^, and p67^phox^), a small GTPase Rac1/2, and membrane subunits (gp91^phox^, and p22^phox^). NOX1 is constituted of the NOX1 catalytic subunit (a homolog of gp91^phox^), NOXO1 (a homolog of p47^phox^), NOXA1 (a homolog of p67^phox^), and Rac1 subunit. The structure of NOX3 is similar to NOX1/2. However, NOX4 constitutes membrane subunits p22^phox^, and poldip2 is significantly different from other NOXs. NOX5 boasts a special N-terminal domain that harbors four Ca^2+^ binding sites and an EF-hand domain. The DUOX1/2 has a unique N-terminal domain and EF hand-type Ca^2+^-binding pockets. The activation of NOX1-3 needs cytosolic subunits, while NOX4 requires p22^phox^ and poldip2. Ca^2+^ that binds to the EF-hand domains is demanded in the activation of NOX5 and DUOX1/2

Mitochondria are the metabolic centers of cells, play an essential role in various fundamental organismal processes, and serve as integral participants in the regulation of cell signaling pathways. For instance, they are the major site of adenosine triphosphate (ATP) generation by oxidative phosphorylation (OXPHOS), as well as participating in ROS generation and consumption, heme synthesis, the tricarboxylic acid (TCA) cycle, calcium signaling, epigenetic regulation, mitophagy, and apoptosis [58, 59]. Hematopoietic stem cells (HSCs) are also regulated by these mitochondrial processes (Fig. 3a). Mitochondrial ROS (mROS), a consequence of electron leakage during OXPHOS and molecular O_2_ reduction, are typical byproducts of mitochondrial respiration [60, 61]. It is well known that mROS encompass a series of major ROS, such as highly reactive superoxide radicals (O_2_^⋅−^), noxious hydroxyl radicals (OH^⋅^), H_2_O_2_, and singlet molecular oxygen (^1^O_2_). It is worth mentioning that there at least ten sites for O_2_^⋅−^ generation in mitochondria, and the complexes I, II, and III of the ETC are most conspicuous [62, 63]. Incomplete electron transport via ETC complexes I and II leads to the generation of O_2_^⋅−^ in the mitochondrial matrix, as well as production in both the mitochondrial matrix and the intermembrane when electronic leakage occurs at complex III [64, 65]. Under pathological conditions, complex III-generated O_2_^⋅−^ commonly results from hypoxic signaling and the activation of hypoxia-inducible factors [66]. In addition, the intermembrane space O_2_^⋅−^ is highly likely to engage in cellular signals transduction events, such as DNA and protein modifications, as they can travel to the cytosol easily [62, 67, 68].

To maintain redox homeostasis within the mitochondria, superoxide radicals in the cytosol, mitochondrial matrix, or intermembrane space are rapidly biotransformed to H_2_O_2_ in the presence of SODs. As noted above, H_2_O_2_ decomposes to form OH^⋅^ via the Fenton reaction, and the elimination of OH^⋅^ is regulated by catalase and peroxidase in the Trx and GSH antioxidant systems. Approximately 90% of intracellular ROS are produced by mitochondrial metabolism; mROS and related signaling pathways are integral participants in a diverse array of processes, including senescence, apoptosis, tumorigenesis, and development [69]. Previous studies have reported the critical role of mitochondria and ROS in self-renewal [70, 71], differentiation [72], fate [73], and function [74].

### NADPH oxidase (NOX) family proteins

NOX are multi-subunit protein complexes that belong to the NOX family. The classical NOX structure is composed of two membrane catalytic subunits (gp91^phox^ referred to as NOX2 and p22^phox^), three cytosolic proteins (p47^phox^, p40^phox^, and p67^phox^), and the G-protein *Rac* [75]. Seven NOX isoforms have been identified, including NOX2 and its homologs (NOX1, NOX3, NOX4, and NOX5), dual oxidase 1 (DUOX1), and dual oxidase 2 (DUOX2) [76]. The NOX family was initially discovered in the phagocytic membrane, and NOX2 was the first identified member of this family. NOXs are important for mature phagocytes to exterminate pathogens and regulate immune defense and inflammation [77]. Professional phagocytic cells (neutrophils, eosinophils, monocytes, and macrophages) can use superoxide-produced NOXs as part of the antimicrobial mechanisms to derive large amounts of ROS [75]. The NOX family is one of the major endogenous enzymes that can induce the cellular production of O_2_^⋅−^ and H_2_O_2_ by transferring electrons from the cytosolic donor NADPH to the acceptor O_2_ [75]. They are ubiquitously present in virtually all organs, tissues, and cells, and are closely linked to cellular proliferation and differentiation, aging, apoptosis, and even the pathological mechanisms of many diseases.

The subcellular localization of NOXs is significantly different, which is conducive to local ROS production and cellular signal transduction. For instance, NOX1, NOX2, NOX3, DUOX1, and DUOX2 are mainly found in the plasma membrane (PM); NOX5 is distributed in the endoplasmic reticulum (ER); and NOX4 is observed in the PM, ER, and nucleus (N) [18]. In addition, there are marked distinctions in the tissue distribution of the NOX family proteins: the colon boasts the most abundant expression of NOX1, and NOX2 is primarily expressed in mature phagocytes; concurrently, the most abundant tissues of NOX3, NOX4, NOX5, and DUOX proteins are the inner ear, kidney, spleen and testis, and thyroid, respectively (Table 1) [78, 79].

**Table 1:** Summary of major ROS generators in hematopoietic cells

Name	Location	Product	Tissue distribution
Mt ETC	Mitochondria	O_2_^⋅−^	Ubiquitous
NOX1	PM	O_2_^⋅−^	Colon epithelial cells, vascular smooth muscle, endosome
NOX2 (gp91^phox^)	PM, ER, ES	O_2_^⋅−^	Phagocytes, lymphocytes, neurons
NOX3	PM	O_2_^⋅−^	Inner ear, fetal kidney, spleen
NOX4	ER, PM, nucleus	H_2_O_2_	Kidney, endosome, vascular endothelial cells, vascular smooth muscle
NOX5	ER	O_2_^⋅−^	Spleen, testis, vascular smooth muscle
DUOX1	PM	H_2_O_2_	Thyroid, lung, prostate, testis, salivary glands, pancreas
DUOX2	PM	H_2_O_2_	Thyroid, intestinal tract
XOR	Cyto, ExC	O_2_^⋅−^	Liver, intestine, mammal gland
MPO	PM	HOCl	Bone marrow, phagocyte
CYP3A4	ER	O_2_^⋅−^/H_2_O_2_	Liver, small intestine, duodenum
CYP2D6	ER	O_2_^⋅−^/H_2_O_2_	Liver, small intestine
CYP2E1	ER, M	O_2_^⋅−^/H_2_O_2_	Liver
CYP4A11	ER	O_2_^⋅−^/H_2_O_2_	Kidney, liver

Some NOXs (NOX1–4) require association with the transmembrane subunit p22^phox^ to ensure correct posttranslational modification, membrane targeting, long-term stability, and enzymatic activity; however, the structures and regulatory mechanisms of the seven enzymes greatly vary (Fig. 3b). NOX1 activation, which leads to the reduction of O_2_ to superoxide, is completed by forming a complex with NADPH oxidase activator 1 (NOXA1), NADPH oxidase organizer 1 (NOXO1), and Rac1 GTPase [80]. The activation of NOX2 is regulated by a set of complex protein–protein interactions (p22^phox^, p47^phox^, p67^phox^, p40^phox^, and Rac), as previously reported [81]. Intriguingly, both the cytosolic subunits (p47^phox^ and p67^phox^) and activators (NOXA1 and NOXO1) can mediate NOX3 activation and are required for the p22^phox^ subunit in NOX3 activation and superoxide formation [82]. It is distinct from other NOXs. NOX4 with compositional activity does not require cytoplasmic subunits to function, and it merely hinges on the p22^phox^ protein for ROS production. Studies have shown that NOX5 activation is mediated by the intracellular Ca^2+^ concentration, as it boasts a special N-terminal domain that contains Ca^2+^-binding pockets that prompt NOX5 activation by extra elongation factor (EF)-hand motifs [75, 83]. Similar to NOX5, DUOX1/2 protein activation is dependent on Ca^2+^ because their structures have additional N-terminal domains with peroxidase activity and intracellular EF hand-type Ca^2+^ binding sites [84]. Additionally, dual oxidase maturation factors play a paramount role in the posttranslational modification and membrane targeting of DUOX1/2 [78]. Although each member of the NOX family produces ROS, distinct types of ROS are generated. NOX1, NOX2, NOX3, and NOX5 mainly produce highly reactive O_2_^⋅−^ and NOX4, whereas DUOX1/2 enzymes principally generate H_2_O_2_ (Table 1).

The NOX family has attracted considerable attention because of its involvement in the pathogenesis and progression of numerous diseases, including various neoplasias. In particular, the redox signaling molecule H_2_O_2_, which originates from the NOXs, plays a critical role in hematopoiesis [85] and hematopoietic growth factor signaling [86]. Hole et al. [87] reported that the constitutive activation of NOXs caused the generation of extracellular ROS to be significantly augmented in more than 60% of patients with AML and that the increased ROS prompted the proliferation of AML cells, as well as normal CD34^+^ cells, to a lesser extent. Demircan et al. [88] analyzed the important role of the NOX family member NOX4 in AML using human AML cells and mouse models. They revealed that the proliferation ability and cell competition were reduced in fms-like receptor tyrosine kinase 3-internal tandem duplication (FLT3-ITD)-positive human AML cells upon inhibition of the enzymatic activity of NOX4 and p22^phox^. In contrast, an augmented myeloproliferative phenotype was observed in the FLT3-ITD-triggered knock-in mouse model after the deliberate deletion of NOX4. Meanwhile, NOX4 inactivation leads to increased HSC numbers, and the reconstitution ability decreases slightly in normal hematopoietic stem and progenitor cells (HSPCs).

### Metabolic pathways

Specific metabolic pathways or enzyme activities are associated with cellular ROS generation, including polyamine metabolism, purine catabolism, activities of xanthine oxidoreductase (XOR), myeloperoxidase (MPO), and cytochrome P450 (CYP) monooxygenase enzymes. Mounting evidence strongly indicates that tumors contain higher levels of polyamines and amine oxidases (AOs) than normal tissues; using primary amines as electron donors, AOs can catalyze polyamines to form aldehydes, ammonia, and H_2_O_2_ in response to cellular signals and stress [89]. XOR is a rate-limiting enzyme that converts hypoxanthine to xanthine and xanthine to uric acid [90]. Two interconvertible forms of XOR are possible: mammalian XOR, which is constitutively a nicotinamide adenine dinucleotide (NAD^+^)-dependent xanthine dehydrogenase, can be transformed to xanthine oxidase (XO) either reversibly by the oxidation of two cysteine residues or irreversibly by proteolysis [91]. XO catalyzes the reduction of O_2_ to generate O_2_^⋅−^ and H_2_O_2_ by transferring monovalent and divalent electrons [92]. Previous studies have illustrated that XO can be activated by inflammatory stimuli or stem cell growth factors and is essential for the maintenance of mammalian target of rapamycin (mTOR)-dependent translational regulation in human myeloid cells [93]. Moreover, XOR-derived ROS can induce OS and enhance the interactions between leukocytes and endothelial cells by increasing phagocytic adhesion [92].

MPO is a heme peroxidase that mainly exists in primary azurophilic granules, whereas very small amounts of MPO are found in monocytes and certain macrophage subpopulations. MPO can catalyze chlorides to form hypochlorous acids that participate in other types of ROS production, including OH^⋅^ and NO_2_Cl [10]. Notably, a recent study has reported that the expression level of MPO strongly interferes with the sensitivity of AML cells to cytarabine and plays a pivotal role in maintaining mitochondrial metabolism and redox homeostasis [15]. The measurement of neutrophil MPO expression in peripheral blood can effectively exclude patients with suspected MDS [94]. In addition, CYPs, which are part of the electron transport chain in the ER, are capable of inducing ROS generation upon breakdown or uncoupling of the P450 catalytic cycle [95]. As one of the primary sources of ROS, CYPs play a significant role in the oxidative metabolism of several endogenous and exogenous compounds [96].

## Functions of ROS in the hematological niche

### Basics of the hematological niche

**Fig. 4:**
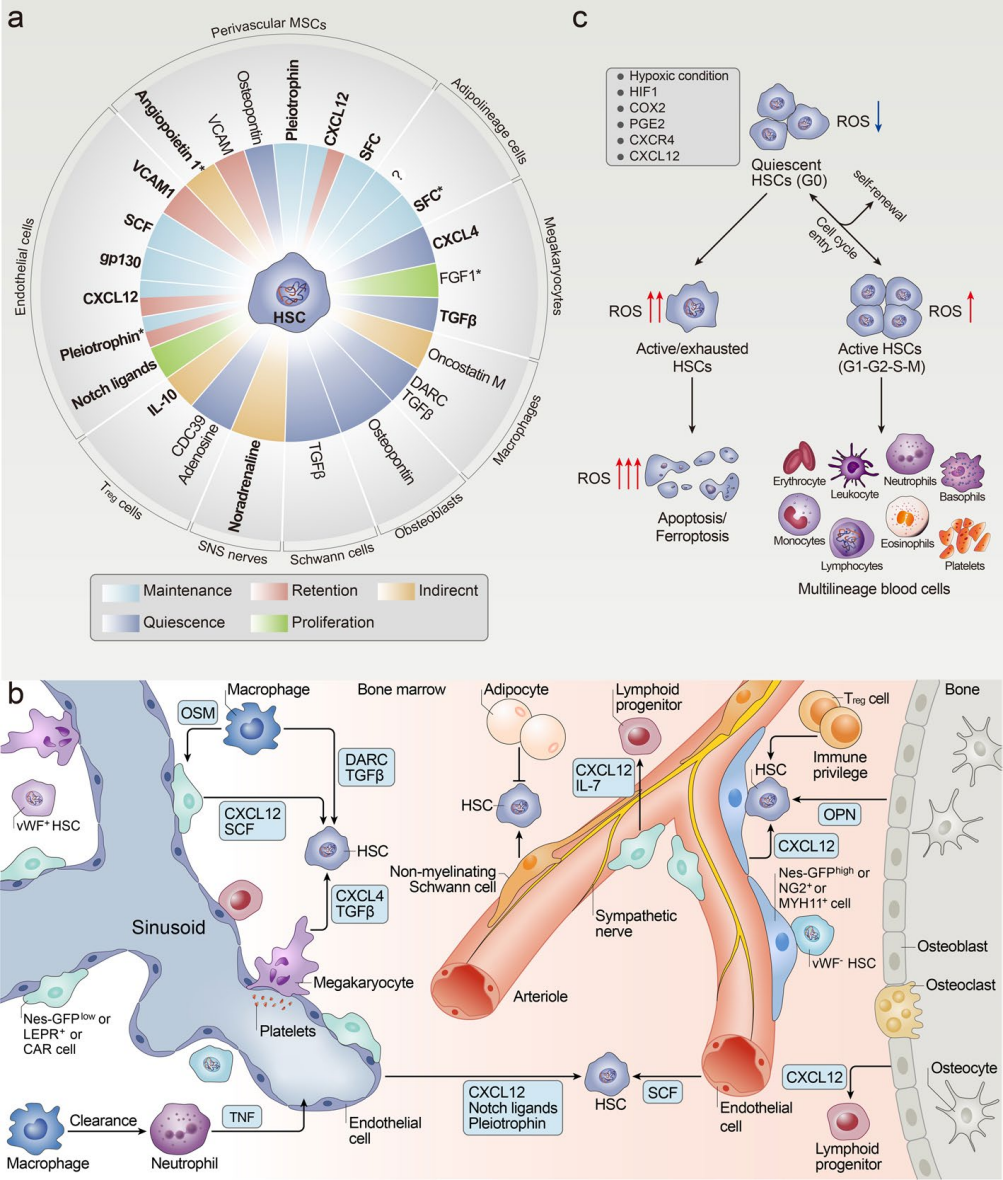
**a** Cellular and molecular components of the HSC niche. The activity of HSC is regulated by various nonhematopoietic and hematopoietic cell types and niche regulatory factors. The target map shows how BM niche cells are indirectly or directly implicated in the regulation of HSCs through the synthesis of niche factors in the form of cell-bounding or secretory molecules. The color of radial spokes represents the affected HSC activity. Molecules with asterisks stand for involvement in BM regeneration after ablation. The bold molecules indicate molecules for which functional data were obtained via cell-specific genetic evidence. **b** The adult bone marrow HSC niche in homeostasis. Multiple cell types and niche regulatory factors are implicated in the regulation of HSC activity in a direct or indirect manner. Vasculature and associated stromal cells, including periarteriolar *Nes*-GFP^high^ cells, NG2^+^ cells, and MYH11^+^ cells, as well as perisinusoidal *Nes*-GFP^low^ cells, CAR cells, and LEPR^+^ cells, are the essential regulators for HSC maintenance. The sympathetic nervous system nerves are involved in the mobilization of HSC, adipocytes perhaps negatively impact HSC maintenance, and nonmyelinating Schwann cells may lead to HSC quiescence. Osteoblasts not only take part in HSC regulation but also may play a prominent role in lymphoid progenitor regulation. Macrophages, neutrophils, T_reg_ cells, megakaryocytes, and other hematopoietic cells are the progeny that differentiate from HSC. In addition, platelet-biased *Vwf*-GFP^+^ HSCs are distributed in and regulated by separate BM niches containing megakaryocytes, while myeloid-biased *Vwf*-GFP^–^ HSCs are localized in and regulated by separate BM niches containing arterioles. **c** The relationship between ROS levels and HSCs destiny. Maintenance of low ROS levels is associated with hypoxic conditions and some regulators, such as HIF1, COX2, PGE2, CXCR4, and CXCL12. Raised ROS could drive HSCs out of the quiescent state and differentiation into short-term repopulating cells, and further differentiation into myeloid cells (e.g., erythrocytes, leukocytes, neutrophils, basophils, eosinophils, monocytes, lymphocytes, and platelets). However, excessive ROS levels can prompt the exhaustion of HSCs and then apoptosis/ferroptosis

In the bone marrow (BM), HSCs and progenitor cells dwell within the so-called hemopoietic niche, which is defined as cellular and molecular microenvironments that ensure hematopoietic homeostasis, maintenance and regulation of HSC functions, control of their normal growth, self-renewal proliferation and differentiation, and migration through the collaboration of cellular mechanisms. Typically, the hematological niche is divided into two distinct compartments, viz. the osteoblastic marrow compartment and the vascular marrow compartment, which are essential for hematopoiesis [97]. On the basis of transgenic mouse models, various BM stromal, nonhematopoietic and hematopoietic cell types, niche factors, and their receptors have been implicated in the regulation of intricate hemopoietic niche activity (Fig. 4a) [98, 99].

Mature blood cells originate from a population of pluripotent HSCs that are mostly quiescent while sporadically dividing and self-renewing to sustain the stem cell pool and ensure continued blood cell replenishment [58, 100]. The growth of HSCs can be divided into two phases: quiescent (cell cycle in the G0 phase) and activated (cell cycle in the G1–G2–S–M phase). Notably, during cell cycle progression triggered by elevated intracellular ROS levels, activated HSCs can not only choose to proliferate and differentiate to form multilineage blood cells but also reenter a quiescent state. The anatomical location of endogenous HSCs is mainly adjacent to sinusoidal blood vessels and away from arterioles after activation and proliferation [101, 102], whereas quiescent HSCs are located in proximity to megakaryocytes and osteoblastic cell compartments [103, 104]. The distribution of HSCs in hematological niches may not be random and is likely affected by the anfractuous cellular and molecular microenvironments in the BM. It is becoming increasingly apparent that a variety of BM stromal cells, HSCs’ progeny, and other cell types are involved in the regulation of HSC activity. Endothelial cells, perivascular mesenchymal stromal cells (MSCs), adipose cells, and macrophages can produce stem cell factor, CXC-chemokine ligand 12 cytokines, and other regulatory factors that promote HSC self-renewal and are required for HSC maintenance [105–108]. Crosstalk between nonhematopoietic and hematopoietic cell types and niche regulatory factors ensures optimal growth of HSCs (Fig. 4b) [109].

### Fate of ROS and HSCs

In addition to the various cell types and regulatory factors in the BM niche microenvironment, intracellular ROS are also implicated in the regulation of HSC activity. Several studies have elucidated the prominence of ROS management in HSC functions, including hematopoiesis, self-renewal, proliferation, differentiation, maturation, migration, and chronological aging. Specifically, HSCs are extremely sensitive to the intracellular redox state; thus, maintaining extremely low cellular ROS levels and NOX expression levels is essential for HSCs to maintain quiescence [110]. Evidence suggests that quiescent, proliferative, and differentiated stem cells boast distinct amounts of intracellular ROS owing to their different metabolism. Low ROS levels, which are regulated by both endogenous and exogenous factors, are required for the maintenance of stem cell self-renewal, migration, and development, and the cell cycle state [111, 112]. However, increased ROS seemingly drive HSCs out of quiescence and trigger HSC differentiation, reducing their capacity for self-renewal, disrupting the balance between self-renewal and differentiation, and exhausting the HSC pool if not remedied, which, in turn, may promote the onset of certain types of disease [71, 97, 113, 114]. Therefore, intracellular ROS levels may determine the fate of stem cells (Fig. 4c).

### Regulation of hematopoietic homeostasis

Numerous scientific studies have shown that abnormal differentiation and self-renewal of HSC can cause certain types of diseases. For example, MDS or leukemia results from insufficient differentiation or uncontrolled self-renewal of HSC, whereas excessive differentiation or insufficient self-renewal can contribute to the depletion of the HSC pool [99]. To maintain hematopoietic homeostasis throughout the life cycle, the differentiation, self-renewal, and aging of HSCs must be regulated. The forkhead box O (FOXO) family of transcription factors (especially FOXO3), which serve as crucial regulators of ROS levels in cellular antioxidative defense systems, is essential for maintaining the HSC pool [115–117]. Yalcin et al. [118] studied FOXO3(−/−) mice and demonstrated that FOXO3 regulation of HSC occurs mostly by regulating the redox state of HSC, in which the loss of FOXO3 leads to elevated ROS accumulation and myeloproliferative syndrome that can be partially rescued by antioxidant therapy. Furthermore, FOXO3 loss in HSCs reduced the competitive repopulation ability and induced exhaustion of the HSC pool in an in vitro model [115]. Importantly, previous studies have shown that FOXO3 is involved in the regulation of mitochondrial metabolism in HSCs [119–121]. These findings indicate that FOXO3(−/−) HSCs can cause fragmented mitochondria, increased mitochondrial content, mitochondrial membrane potential (MMP), and glycolysis, but reduced OXPHOS and ATP; the mitochondrial defects of HSC (rather than increased ROS levels) are associated with the long-term competitive repopulation activity of HSCs. Additionally, these studies also singled out the possibility that enhanced activity of glycolysis may have a bearing on exit from quiescence and HSC activation in at least some contexts [119], although the majority of literature has revealed that normal HSCs reside in a low-oxygen niche environment and their energy demands are highly dependent on the glycolytic pathway [122–124].

In addition to *FOXO3*, several other genes participate in the regulation of mitochondrial metabolism and affect the function and fate of HSCs. Maryanovich et al. [125] demonstrated that the ataxia–telangiectasia mutated (ATM)-mediated BH3 interacting domain death agonist (BID) pathway plays a critical role in the self-renewal and quiescence maintenance of HSCs by regulating OS. Loss of BID phosphorylation results in HSC escape from the quiescent phase, HSC pool depletion, and a significant reduction in HSC reproductive potential. In parallel, they found that the mitochondrial carrier homologue 2 (MTCH2), downstream of BID, negatively regulates mitochondrial OXPHOS and is indispensable for HSC homeostasis. Loss of MTCH2 enhances the mitochondrial size and OXPHOS, increases ATP and ROS levels, and triggers HSPC cycle entry [126]. Tai-Nagara et al. [127] demonstrated that mortalin and DJ-1 act synergistically and are imperative for HSCs to maintain normal physiological ROS concentrations and HSC numbers. Furthermore, a study on the tuberous sclerosis complex (TSC)/mTOR signaling pathway showed that HSCs with TSC1 deletion escaped quiescence and mitochondrial biogenesis, as well as a marked reduction in hematopoiesis and self-renewal capability [128]. TSC1(−/−) activates mTOR signaling in response to ROS generation in HSC. These findings indicate that mitochondrial metabolism and intracellular ROS levels are important regulators of HSC function and must be precisely regulated.

## Pathophysiology of MDS

### Classification of MDS

MDS is a hematological neoplasm with limited treatment strategies, being characterized by clonal propagation of HSCs, recurrent genetic abnormalities, myelodysplasia, ineffective hematopoiesis, abnormalities in the peripheral blood, and a high intrinsic risk of progression to AML [129, 130]. Patients with MDS have been stratified into five risk groups according to the revised International Prognostic Scoring System (IPSS-R), including IPSS-R very low, low, intermediate (up to 3.5 IPSS-R score points), high, and very high risk, with distinct clinic outcomes in terms of survival and AML evolution [131]. Recently, the World Health Organization and the International Consensus Classification have updated the latest classification of MDS, which is in favor of more holistic risk-stratification schemes (e.g., IPSS-R). Notably, the new classification divides MDS entities into those with well-defined genetic abnormalities and those with morphological definitions, and places more emphasis on defining MDS typing from a genetic perspective than the previous version of risk-based typing (Table 2) [132, 133].

**Table 2:** The latest classification and defining characteristics of MDS [132]

	Blasts	Cytogenetics	Mutations
MDS with defining genetic abnormalities			
MDS-5q (MDS with low blasts and isolated 5q deletion)	< 5% BM and < 2% peripheral blood	5q deletion alone, or with one other abnormality other than monosomy 7 or 7q deletion	
MDS-*SF3B1* (MDS with low blasts and SF3B1 mutation)	< 5% BM and < 2% peripheral blood	Absence of 5q deletion, monosomy 7, or complex karyotype	*SF3B1*
MDS-bi*TP53* (MDS with biallelic TP53 inactivation)	< 20% BM and peripheral blood	Generally complex	Two or more TP53 mutations, or one mutation with evidence of TP53 copy number loss or copy neutral loss of heterozygosity
MDS, morphologically defined			
MDS-LB (MDS with low blasts)	< 5% BM and < 2% peripheral blood		
MDS-h (hypoplastic MDS)			
MDS-IB (MDS with increased blasts)			
MDS-IB1	5–9% BM or 2–4% peripheral blood		
MDS-IB2	10–19% BM or 5–19% peripheral blood or Auer rods		
	5–19% BM; 2–19% peripheral blood		

### Molecular pathogenesis of MDS

**Fig. 5:**
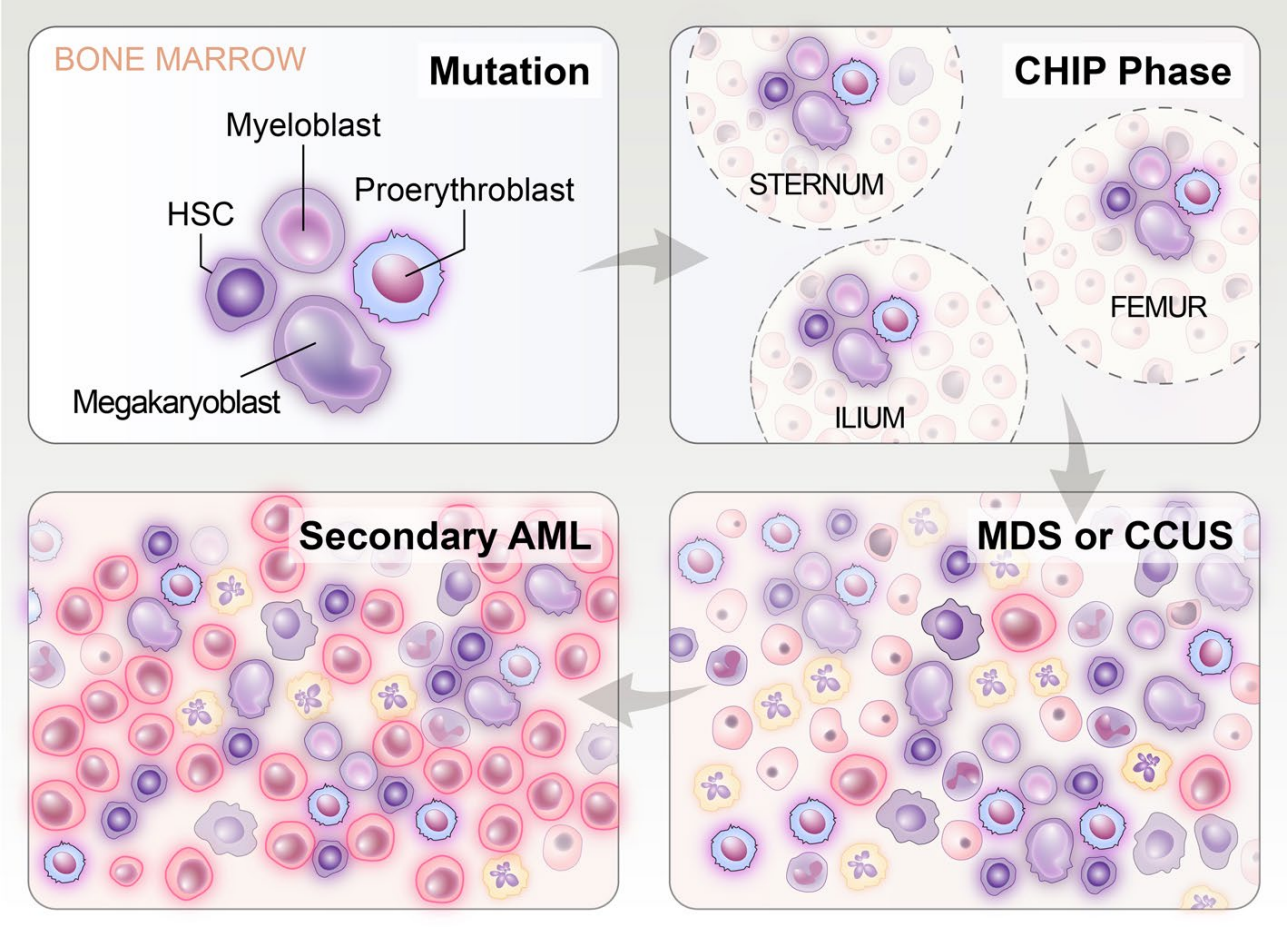
Occurrence and manifestations of myelodysplastic hematopoiesis. MDS develops from the growth and propagation of a clone with somatic mutations of hematopoietic cells and generally evolves into AML. The characteristics and clinical manifestations vary in different phases. First, an initial mutation occurs in HSC, and additional mutations that pertain to clonal progression occur in progenitor or precursor cells, collectively forming a local clone. Next, as time elapses, mutant stem cells migrate and dwell within other BM regions (e.g., sternum, femur, and ilium) through peripheral blood to form local clones, and the condition is defined as the clonal hematopoiesis of indeterminate potential (CHIP) phase when hematopoietic cells harboring somatic mutations represent a minimum of 4% of all BM cells (corresponding to a minimum of 2% of the mutation allelic frequency). Subsequently, clonal hematopoiesis gradually increases and ultimately becomes the predominant cell population in the BM, which is called MDS or clonal cytopenia of undetermined significance (CCUS). The abnormal hematopoiesis caused by clonal dominance is frequently linked to additional somatic mutations. Ultimately, the emergence of additional driver mutations acquirement or preexisting mutations results in the selection and leukemic transformation of subclones of hematopoietic cells (highlighted in pale pink) with progressively damaged capacity for differentiation

MDS develops from the growth and spread of a clone with somatic mutations of hematopoietic cells and generally evolves into AML (Fig. 5) [129]. The selection advantage of clones is conferred by somatic genetic lesions described as driver mutations [134], and the initial mutation takes place in HSCs with self-renewal capability. Meanwhile, additional mutations that pertain to clonal progression may also occur in progenitor cells, thereby bestowing the ability to self-renew [135]. Some mutation driver genes that belong to distinct biological pathways can contribute to myelodysplastic neoplasm, and the majority of patients with MDS exhibit combinations of pathway mutations, which is responsible for the heterogeneity of MDS [136–139].

#### Pathophysiology of MDS with isolated del(5q)

MDS with isolated del(5q) is caused by the deletion of the DNA region in the long arm of chromosome 5. This genetic lesion is the initial driver mutation that results in the haploinsufficiency of several genes, which subsequently drives clinical symptoms. Typically, ribosomal protein S14 (RPS14) and casein kinase 1 alpha 1 (CSNK1A1) are associated with the dysplasia of erythrocytes, and RPS14 haploinsufficiency contributes to macrocytic anemia in mutant erythroblasts [140]. CSNK1A1 haploinsufficiency is capable of endowing del(5q)-heterozygous stem cells with clonal growth superiority and then expansion [141], which is responsible for the efficiency and high clinical remission rate of lenalidomide in MDS with isolated del(5q) [142].

#### Mutation driver genes

In MDS patients, there are numerous mutation driver genes, which, through diverse mechanisms, lead to clonal outgrowth, myeloproliferation, and propagation of myelodysplastic hematopoiesis (Fig. 5). Only ASXL transcriptional regulator 1 (ASXL1), DNA methyltransferase 3 alpha (DNMT3A), RUNX family transcription factor 1 (RUNX1), splicing factor 3b subunit 1 (SF3B1), serine and arginine rich splicing factor 2 (SRSF2), and tet methylcytosine dioxygenase 2 (TET2) exhibit mutations in a minimum of 10% patients [138, 139, 143]. The most frequently mutated genes in MDS, TET2, and DNMT3A are essential for the differentiation of HSCs [144]. The heterozygous inactivation of TET2 augments self-renewal and damage differentiation, resulting in clonal growth of mutant stem cells and myeloproliferation [145]. DNMT3A ablation in the hematopoietic system leads to myeloid transformation, affecting stem cell self-renewal, myeloid differentiation, tissue tropism, and restricting progenitor expansion [146].

#### Abnormality of RNA splicing and aberrant gene transcripts

**Fig. 6:**
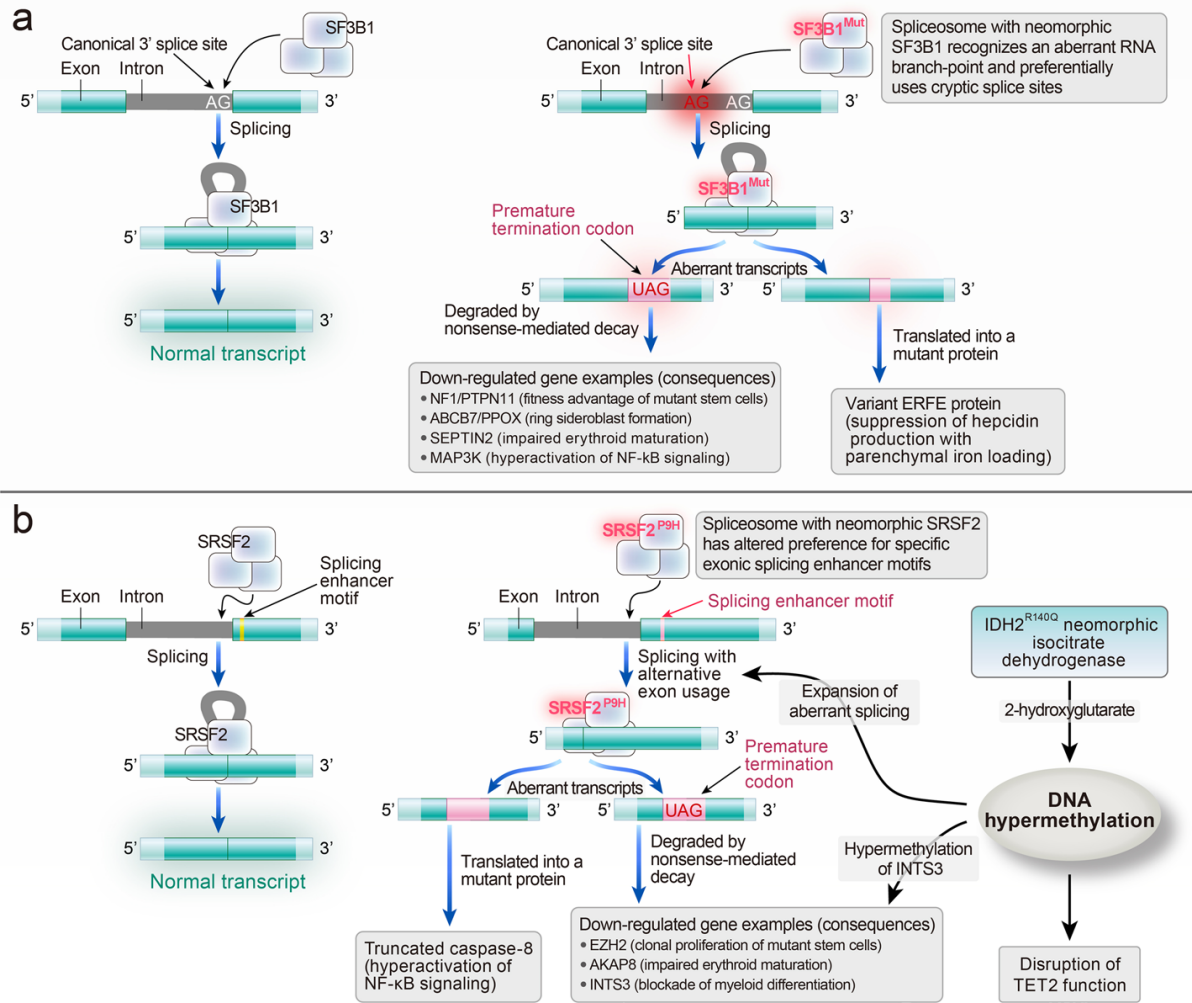
**a** Role of abnormality of RNA splicing in the pathogenesis of SF3B1-mutated MDS. In hematopoietic cells, normal spliceosomes implement roughly half of the splicing events, while the other half is performed by spliceosomes containing a mutant SF3B1 splicing factor, which alters the recognition of RNA branch points and renders the preferred usage of cryptic 3′ splice sites located 10–30 base pairs farther upstream of canonical sites, finally causing aberrant transcripts of several genes or in-frame isoforms production. However, just small quantities of abnormal transcripts are detectable in SF3B1-mutated myelodysplastic cells owing to the bulk of abnormal transcripts’ rapid degradation through nonsense-mediated decay, which is primarily caused by the inserted nucleotide sequence containing a premature termination codon. **b** Synergistic interaction of aberrant splicing and epigenetic dysregulation in MDS. Mutation of SRSF2 renders the preference alteration of the neomorphic splicing factor to specific exonic splicing enhancer motifs, in turn causing alternative exon usage. Aberrant transcripts with a premature stop codon will be generated in the process, with rapid degradation through nonsense-mediated decay or the production of mutated proteins, resulting in different pathological outcomes. Mutation of IDH2 (R140Q) gives rise to the activation of the neomorphic enzyme and, in turn, DNA hypermethylation, which sabotages epigenetic regulators’ function and drives the malignant advancement of the disease

According to recent studies, SF3B1 mutation has been identified as a unique subtype of MDS that encompasses more than 90% of MDS cases with ineffective erythropoiesis, and at least 5% ring sideroblasts [147, 148]. In proven cases, specific mutations or comutations and the amount and type of mutations mostly tend to be unfavorable to the prognosis of MDS patients, with certain exceptions where SF3B1 mutation confers a superior outcome and prolonged survival [148–150]. In hematopoietic cells, roughly half of the splicing events are performed by spliceosomes containing a mutant SF3B1 splicing factor, which alters the recognition of RNA branch points and renders the preferred usage of cryptic 3′ splice sites, finally causing aberrant transcripts of several genes or in-frame isoform production (Fig. 6a) [151–153]. The situation involves erythroferrone (ERFE): variant ERFE protein is conducive to increased iron absorption or parenchymal iron loading [153]. Furthermore, mutation driver genes SRSF2, U2 small nuclear RNA auxiliary factor 1 (U2AF1), and the epigenetic regulator isocitrate dehydrogenase 1 and 2 (IDH1/IDH2) are recurrently mutated in numerous myeloid neoplasms and are associated with unfavorable clinical prognosis [154–159]. Compared with spliceosome gene SF3B1 mutations, SRSF2 and U2AF1 mutations result in different splicing abnormalities, mainly alterations in exon usage [151, 152, 160]. Their mutation is concerned with augmented R-loop formation, which results in genomic instability and is always associated with combinatorial mutation patterns [152, 161], such as the comutation of SRSF2 (P95H)–IDH2 (R140Q) found in MDS and AML [138, 162, 163]. Collectively, the interaction between abnormality RNA splicing and epigenetic regulation control drives the malignant advancement of MDS or AML (Fig. 6b).

### ROS in the pathophysiology of MDS

Substantial literature supports that ROS play a paramount role in the occurrence of numerous diseases, as they take part in the regulation of essentially all aspects of cellular function (gene or protein expression, cellular growth, proliferation and differentiation, and epigenetic modifications) [9]. More recently, enhanced ROS levels have been observed in a wide variety of pathological states, such as neurodegenerative, autoimmune, cardiovascular, and metabolic diseases [21, 28, 164, 165], atherosclerosis [166, 167], cataracts [168], Fanconi anemia (FA), and hematological malignancies such as MDS, and AML. Notably, patients with FA frequently develop MDS or AML. In this subsection, we focus on the role of ROS in MDS development.

#### Generation of ROS in MDS

Pioneering studies have found that the presence of oxidized pyrimidine nucleotides in the CD34^+^ cells of patients with MDS and the oxidized pyrimidines were closely related to increased plasma tumor necrosis factor-α and low concentration of GSH in BM mononuclear cells [169]. It is currently clear that the oxidized purine and pyrimidine nucleotides (DNA oxidative damage) are ubiquitously present in the BM CD34^+^ cells of patients with MDS when compared with controls [170], and enhanced ROS levels and oxidative damage markers are also commonly detected. Furthermore, increased activity of several antioxidant enzymes and reduced GSH levels have been observed in patients with MDS [56, 171]. These observations suggest that these patients were under OS.

A plethora of studies suggest that inflammation and the inflammasome, pyroptosis, ferroptosis, mitophagy, and even necroptosis are inextricably linked with ROS generation and affect the pathophysiology of MDS [172–174]. It is becoming more widely recognized that inflammation is a characteristic of MDS, and previous studies have confirmed that activation of the NLR family pyrin domain containing 3 (NLRP3) inflammasome is redox dependent as well as a hallmark of patients with MDS, which causes clonal expansion and pyroptosis upon activation [175, 176]. Specifically, there is excessive protein content of alarmin S100A9 in MDS HSPCs and BM plasma, and S100A9 is capable of triggering pyroptosis through the activation of NOX, augmenting ROS levels, and the activation of NLRP3 and β-catenin. Meanwhile, knockdown of or pharmacologically inhibiting NLRP3, neutralizing S100A9, can alleviate pyroptosis, ROS accumulation, and nuclear β-catenin in MDS, rendering restoration of colony-forming capacity and efficient hematopoiesis [176]. Cluzeau et al. [177] reported that S100A9 directly inhibits the elaboration of erythropoietin and the endocrine response to anemia, while neutralization or suppression of S100A9 could reverse the processes and thus erythropoiesis enhancement in patients with low-risk MDS (LR-MDS). Ji et al. [178] showed that pathologic levels of tumor necrosis factor-alpha and interleukin 6 suppressed erythroid colony formation and drive ineffective erythropoiesis via ROS-induced caspase-3 activation and apoptosis in a double knockout of mDia1 and mir-146a mouse model (mimicking del(5q) MDS). Emerging data indicate that decitabine treatment causes ROS to augment, GSH depletion, GPX4 reduction, and subsequently ferroptosis and necroptosis in MDS cells, and these results are also confirmed in iron overload (IOL) MDS mouse models [179]. Ferroptosis or necroptosis induced by decitabine can be abrogated by ferroptosis or necroptosis inhibitors. Crucially, iron chelators also enhanced the effects of decitabine, indicating that ROS is an essential regulator of treatment outcomes.

Mitophagy is an evolutionarily conserved intracellular process that obviates dysfunctional mitochondria to avoid their accumulation and is eminent in tumorigenesis and treatment [180]. Caspase-dependent apoptosis, ROS-induced mitophagy/autophagy, and accumulation of DNA and mitochondrial damage have been well demonstrated in MDS [181, 182]. Studies indicate that mice manifest loss of HSC functions, myeloproliferation, augmented mitochondria and ROS in the HSPC compartment, and elevated DNA impairment when conditionally deleting autophagy related 7 (Atg7) in the hematopoietic system, indicating that Atg7 is a crucial modulator of HSC maintenance [183, 184]. Additionally, Jiang et al. [185] observed that impairment in NIX-mediated mitophagy is linked to the accumulation of ROS and damaged mitochondria in BM nucleated RBC of MDS patients. Experiments in MDS mouse models showed elevated ROS levels caused by dysregulated mitochondrial dynamics. To be specific, Aoyagi et al. [186] reported that substantial dynamin-related protein 1 (DRP1)-dependent mitochondrial fragmentation in HSPCs results in excessive ROS generation, inducing inflammatory signaling activation and ineffective hematopoiesis, which can be attenuated via DRP1 inhibition. Deactivation of DRP1 in mitochondria can contribute to loss of regenerative potential of HSCs while maintaining their quiescent state [187]. In addition, mitochondrial DNA mutations that are tightly entangled with poor ETC function and increased ROS levels are commonly detected in MDS. The importance of necroptosis in the pathogenesis of MDS has been emphasized. Montalban Bravo et al. [174] reported that receptor interacting serine/threonine kinase 1 (RIPK1, a member of the necroptosis complex component) is highly expressed and associated with poor survival outcomes in MDS patients. Zinkel’s group also presented similar results that necroptosis (predominantly RIPK1 expression) is upregulated in MDS patients compared with control participants [188]. In summary, ROS and OS are capable of inducing cell death (e.g., apoptosis, ferroptosis, pyroptosis, necroptosis, and autophagy) and have been implicated in the pathogenesis and progression of MDS.

#### Inevitable IOL and iron chelation therapy (ICT)

Anemia-related symptoms, such as fatigue, resulting from hematopoietic dysplasia or pancytopenia, commonly occur in most patients with low-risk MDS and lead to red blood cell (RBC) transfusion dependence [189–191], which subsequently results in IOL [192]. IOL is deleterious to cells and can catalyze H_2_O_2_ to easily decompose into highly reactive OH^⋅^ by the Fenton chemistry reaction and are involved in the OS of patients with MDS. Importantly, the accumulation of iron and ROS within BM CD34^+^ cells may contribute to genetic and chromosomal abnormalities, which, in turn, accelerate blast proliferation and prompt MDS transformation into AML [192]. Therefore, it is not surprising that IOL is considered the primary cause of OS in patients with MDS [17, 193]. In addition, IOL is closely associated with the survival outcome of patients with MDS, which negatively affects organ function and clinical survival time [191, 194]. ICT is effective and feasible for the management of patients with MDS and can restore iron balance and improve organ function and survival to near-normal levels, particularly in patients with LR-MDS who are IOL [194–196].

The TELESTO trial found that, compared with placebo, IOL patients with low- to intermediate-1-risk MDS show longer event-free survival without differences in overall survival upon ICT (deferasirox dispersible tablets) [197]. Leitch et al. reported that patients with transfusion-dependent LR-MDS had significantly longer median overall survival time after receiving ICT from the onset of transfusion dependence compared with those who did not [198], and the survival advantage persisted even after conducting a matched pair analysis that accounted for age, frailty, comorbidities, and R-IPSS [199]. Recent studies of 2200 patients with MDS, of whom 224 received ICT, also confirmed that ICT can ameliorate the overall survival and hematopoiesis of transfused patients with LR-MDS. ICT’s benefits for MDS patients vary depending on the circumstances, and National Comprehensive Cancer Network (NCCN) guidelines recommend its usage when ferritin levels surpass 2500 ng/mL [200].

## Is targeting ROS for MDS therapy feasible?

### Possible clinical implications of ROS activity in the hemopoietic system

Hematological malignancies resulting from abnormalities in the hematopoietic system are highly correlated with altered ROS levels. Specifically, ROS are involved in crucial aspects of hematopoiesis, including clonal evolution, hematological improvement, and hematopoietic cell transplantation engraftment. MDS is a well-known clonal disease characterized by elevated genetic instability [136]. In an expanded clone, the continuous acquisition of mutations can first result in a myelodysplastic phenotype and then in a leukemic phenotype through additional mutations [201, 202]. In patients with MDS, IOL can lead to the disruption of ROS homeostasis and genomic instability of pre-leukemia clones, which may be one of the possible reasons for clonal evolution to AML. However, ICT is capable of improving hematopoietic insufficiency in MDS and slowing the progression to AML [192, 193, 203]. In terms of ROS in hematological improvement, studies have revealed that IOL significantly increases ROS levels in HSPCs, reduces the immature hematopoietic cell ratio, and blunts their clonogenic capacity [204, 205]. IOL also increases ROS levels in MSCs of patients with high-risk MDS (HR-MDS) and triggers oxidative injury through the activation of Wnt/β-catenin signaling pathways [206]. Notably, the above effects can be rescued by the administration of iron chelators or antioxidants [206, 207], implying that ROS activity may represent a potential target for therapy. Overall, there is plentiful evidence that excessive free iron adversely affects the hematopoietic microenvironment, resulting in ROS accumulation and affecting the expression of genes that regulate and disrupt hematopoiesis [208]. In addition, several major studies have elucidated the correlation between ROS activity and hematopoietic impairment (Table 3).

**Table 3:** Studies revealing the association between altered ROS levels and damaged hematopoiesis

ROS modulation	Description	Ref.
↑ ROS: augments genomic instability	Excessive iron in MDS patients renders ROS accumulation and then augments genomic instability of the pre-leukemic clone, which accelerates transformation to AML	Pullarkat et al. [280]
↑ ROS: oxidative stress, triggering early hematopoietic cell apoptosis	In an IOL mouse model, leukemic blasts infiltrated the liver and spleen, with fibrosis, extensive necrosis of BM, and massive blast accumulation. Meanwhile, iron is mutagenic and thereby promotes clonal evolution in MDS through DNA damage	Chan et al. [281]
↑ ROS: activation of ROS-related signaling pathway	Heightened ROS levels regulating the expression of redox-sensitive transcription factors (e.g., Nrf2, NF-κB, and HIF1) to prompt leukemogenesis	Zhou et al. [282]
↑ ROS: oxidative stress, DNA double-strand breaks, cell cycle retardation	Based on an MDS murine model, increased ROS levels and mutation frequency in NHD13 BMNCs were observed. In parallel, DNA impairment and oncogenic mutations caused by oxidative stress can expedite the transformation of MDS to AML	Chung et al. [283]
↑ ROS: reduces the ratio and clonogenic function of HSPCs	IOL enhances ROS levels through NOX4 and p38MAPK signaling, thereby affecting the hematopoiesis of BM and the engraftment of HSCs	Chai et al. [207]
↑ ROS: mitochondrial fragmentation and enhanced autophagy in MSCs	IOL contributed to high ROS levels, lowered cell viability and ATP concentrations, mitochondrial fragmentation, and autophagy in MSCs. ICT or antioxidants could deteriorate the change	Zheng et al. [284]
↑ ROS: retards the growth of immature hematopoietic cells	Ferrous ammonium sulfate mediated immature hematopoietic cells’ growth retardation and apoptosis by ROS activation of p38MAPK and JNK pathways, which had negative effects on hematopoiesis	Tanaka et al. [285]
↓ ROS: maintains the self-renewal and multilineage differentiation potential of human HSCs	The small-molecule antioxidant chrysin is able to inhibit ROS-activated apoptosis, and maintain multipotency and long-term activity of hematopoietic stem/progenitor cells	Li et al. [286]
↓ ROS: suppression of apoptosis of hematopoietic stem/progenitor cells	Alpha-lipoic acid can promote HPSC development by upregulating HIF1α in response to a hypoxic environment, also decreasing ROS levels to inhibit HPSC apoptosis	Dong et al. [287]
↑ ROS: inhibits the reconstitution potential of HSPCs	Ionizing radiation caused the rapid and transient increase of ROS and then p38MAPK pathway activation that affects the self-renewal potential of human HSCs	Henry et al. [288]
↑ ROS: retards the proliferation and differentiation of MSCs	IOL results in elevated ROS production and activates Wnt/β-catenin signaling to engage in MDS progression	Huang et al. [206]
↑ ROS: lower membrane potential and DNA damage of SdhcV69E-derived HSCs	Mitochondrial complex II dysfunctions or replicative stresses contribute to white blood cell count decrease, macrocytic anemia, thrombocytosis, as well as ROS accumulation and DNA impairment of HSCs	Harada et al. [289]

### Is there a case for targeting ROS in MDS?

**Fig. 7:**
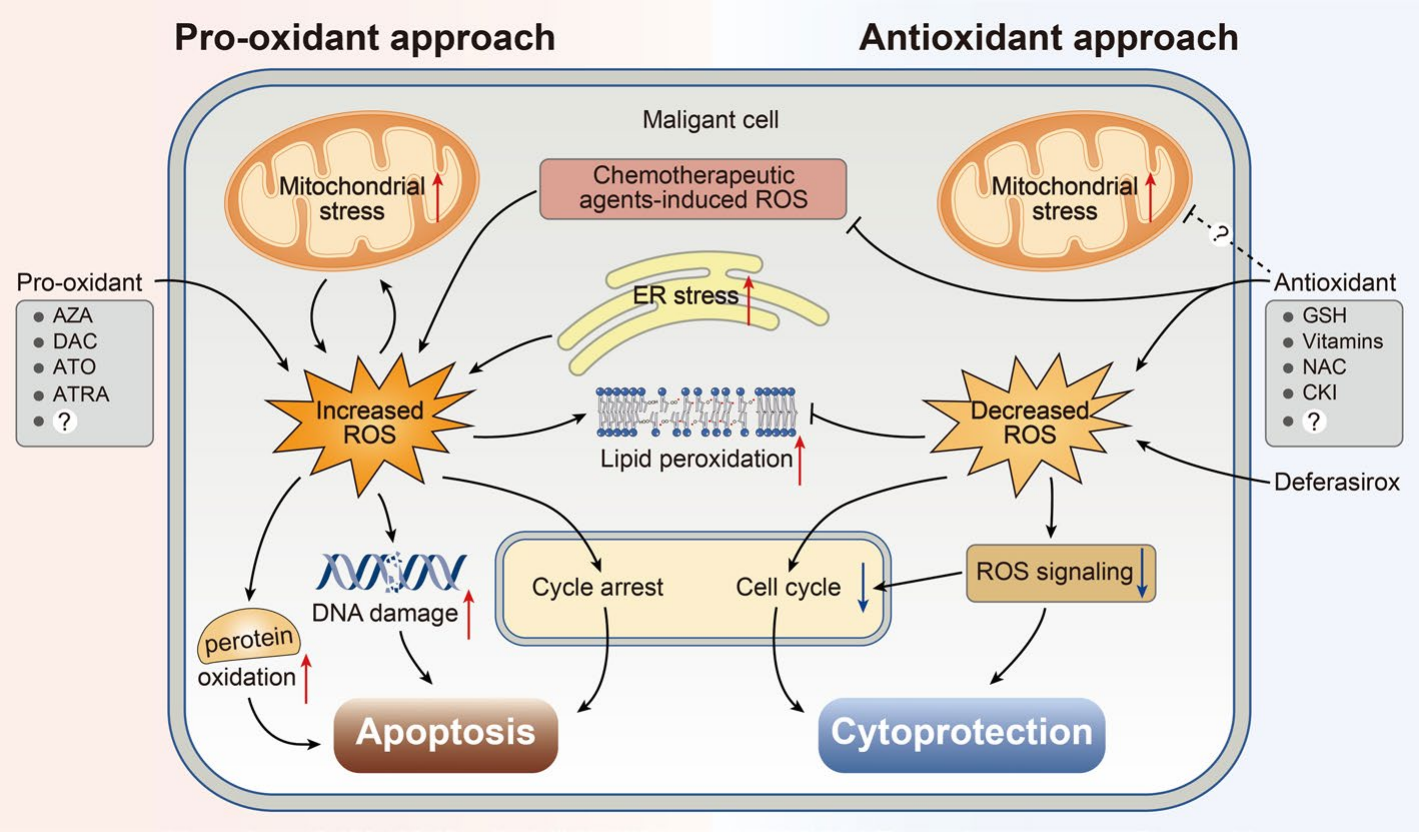
Manipulating ROS levels in MDS cells for therapeutic effects. Schematic representation of prooxidant and antioxidant treatment as a therapy for MDS. The effects of prooxidant treatment are augmented ROS generation, exhausting antioxidant defenses, subsequent unavoidable contributions to oxidative stress, lipid peroxidation, DNA damage, and oxidation of proteins that contain redox-sensitive residues. Moreover, enhanced ROS levels may be conducive to cell cycle progression in some situations, and also promote DNA mutation, which may cause malignant cells to resist apoptosis. However, the application of antioxidants acts against excessive ROS and reduces ROS signaling, oxidative stress, and proliferative drive. In addition, antioxidant treatment could reduce cell cycle progression, and protect nonmalignant cells from oxidative injury, especially when used in combination with chemotherapy

Considering the continued interest in redox chemotherapeutics in recent years [209, 210] and the extensive impact of ROS on MDS pathophysiology and progression, ROS may represent a novel potential therapeutic target for MDS. As discussed above, malignant cells frequently harbor higher ROS levels than their normal counterparts. Increased ROS production leads to hyperactivation of ROS signaling pathways, exhaustion of antioxidant defenses, and nonspecific oxidative damage to biomolecules, particularly DNA and proteins [37]. Therefore, two approaches (prooxidant and antioxidant) can be used to manipulate ROS in malignant cells to achieve therapeutic effects. In this section, we discuss the application of prooxidant and antioxidant approaches in MDS treatment (Fig. 7).

#### The prooxidant approach

The prooxidant approach refers to the amplification of existing oxidative stress and the disruption of redox homeostasis through the administration of prooxidants, which can cause catastrophic oxidative injury and malignant cell death. For many years, cytotoxic drugs have been the mainstay of treatment for hematological malignancies, including MDS and AML. For instance, azacitidine (AZA), which acts as a pyrimidine nucleoside analog of cytidine, disrupts the synthesis of DNA, mRNA, and proteins [211]. Various mechanisms underlie the antineoplastic effects of AZA, such as cytotoxic effects on abnormal hematopoietic cells in the BM, alteration of the cellular redox status, and hypomethylation of DNA [212, 213]. Interestingly, conflicting data exist regarding the effects of AZA on ROS production. A recent study suggested that AZA treatment increases oxidative stress (decreased GSH levels, elevated GSSG·GSH^−1^ ratio in the erythrocyte, and increased lipid peroxidation) in patients with MDS [214]. Klobuch [215] and colleagues showed that low-dose AZA combined with PPARγ agonist pioglitazone and all-*trans* retinoic acid stimulates ROS production and triggers phenotypical and functional differentiation of primary AML blasts into neutrophil-like cells. However, in a case report, Hasunuma et al. [216] observed decreased ROS levels in peripheral white blood cells and reduced dacron-reactive oxygen metabolites (d-ROMs) in the serum of patients with MDS following AZA treatment. The authors concluded that AZA therapy can ameliorate hematopoiesis and weaken ROS and d-ROM generation.

Decitabine (also known as 5-aza-2-deoxcytidine, DAC) is a commonly used drug with Food and Drug Administration (FDA) approval for the treatment of patients with MDS and AML [217]. DAC induces ROS accumulation, cell cycle blockage, and apoptosis in leukemic cells [218–220]. DAC promotes the expression of different NADPH oxidase isoforms and increases the protein expression level of NOX4 in an ATM-dependent manner [221]. Studies conducted by Wang et al. [222] revealed that DAC treatment leads to ROS production, cell growth arrest, MMP reduction, and apoptosis in MSCs derived from patients with MDS. Some chemical compounds with prooxidant properties are effective against MDS and leukemic cell lines. A prototype example is the application of arsenic trioxide (ATO) and all-*trans* retinoic acid (ATRA) in acute promyelocytic leukemia (APL) treatment [223]. The major effect of ATO is the induction of ROS accumulation, which alters cellular redox homeostasis by triggering electron leakage, irreversibly inhibiting thioredoxin reductase, and depleting PRX III [224]. Research has indicated that ATO augments ROS production via Trx inhibition and NOX activation, displaying encouraging results in treatment of relapsed APL [225, 226]. In vitro results from Huang et al. [227] suggested that DAC combined with ATO can induce MDS cell line apoptosis via elevated ROS-related ER stress. Another in vitro study indicated that ATRA blocked the activation of Nrf2 by activating the RARα–Nrf2 complex, rendering ROS accumulation and ROS-dependent cytotoxicity in MDS and AML cells when combined with DAC [228]. Other studies have demonstrated that several drugs can induce MDS cell death by altering cellular ROS levels (Table 4).

**Table 4:** Drugs that alter cellular redox balance in MDS cells

Compound	Results	Ref.
AZA	↑ ROS; ↑ GSSG/GSH; ↑ lipid peroxidation; ↓ GSH	Montes et al. [214]
DAC	↑ ROS; ↓ MMP	Wang et al. [222]
DAC combined with ATO	↑ ROS; ↑ ER stress	Huang et al. [227]
ATO combined with triptolide	↑ ROS; ↑ Bax; ↑ caspase-3; ↓ BCL-2	Hua et al. [290]
DAC combined with ATRA	↑ ROS; ↑ RARα-Nrf2 complex; ↓ Nrf2	Wang et al. [228]
T-dCyd combined with venetoclax	↑ ROS; ↓ Nrf2; ↓ HO-1; ↓ BCL-2	Hu et al. [291]
Withaferin A	↑ ROS; activation of JNK/AP-1 signaling	Oben et al. [292]
Luteolin	↑ ROS; ↑ Bax/BCL‑2; ↑ activity of caspase-3, -8, -9	Dong et al. [293]
Ascorbic acid	↑ ROS; cell cycle arrest	Niu et al. [294]
Fucoidan	↑ ROS; ↑ caspase-3, -8, -9	Wei et al. [295]
DFX	↓ ROS; ↓ DNA oxidative damage	Jiméne et al. [239]

The key role of antioxidants, particularly GSH and Trx, in all cells is to respond to oxidative stress and buffer excess ROS. Thus, inhibition of intracellular antioxidants is sufficient to subvert cellular redox homeostasis and kill tumor cells. Notably, many antioxidant molecules are upregulated in tumor cells, which can influence the therapeutic efficacy and augment drug resistance [49]. Taken together, introducing exogenous ROS or prompting their generation in MDS cells using drugs or chemotherapy may be attractive approaches for MDS treatment.

#### The antioxidant approach

The antioxidant approach aims to scavenge high physiological levels of ROS in some types of cancer using antioxidant molecules. The basic rationale behind this approach is that enhanced ROS accumulation facilitates carcinogenesis and tumorigenesis by inducing gene mutations, increasing genetic instability, and activating prooncogenic signaling [8, 229, 230]. High ROS levels caused by high-glucose conditions can promote the proliferation of pancreatic carcinoma cells [231]. However, there is still a dispute regarding the therapeutic effect of the antioxidant approach in cancer treatment. Some studies have argued that antioxidants protect not only healthy cells but also tumor cells to avoid or reduce oxidative damage, thereby contributing to the effectiveness of chemotherapy being seriously reduced. However, the endorsers believe that antioxidant therapy may counteract chemotherapy-related cytotoxicity, augment treatment response rates, and prolong patient survival. Indeed, several studies have supported antioxidant therapy as a viable option that reduces the toxicity of chemotherapy by damaging malignant cells and does not interfere with chemotherapy when the antioxidant is used concomitantly with chemotherapy [232, 233]. Therefore, it is conceivable to harness an antitumor antioxidant approach with chemotherapy, although the effect of antioxidant therapy in reducing ROS levels has not been widely accepted [234, 235].

Deferasirox (DFX), an iron-chelating drug, is commonly used to treat IOL in patients with LR-MDS [236]. It directly removes labile iron, reduces oxidative stress, improves hematopoiesis, and delays leukemic transformation [237–239]. In addition to this, several exogenous sources of natural or synthetic antioxidants have demonstrated therapeutic potential for tumor treatment. Zhang et al. [240] reported that the antioxidant azelaic acid can reduce ROS levels, elevate the total antioxidant capacity of AML cells, and exhibit antileukemic effects. In leukemic cells, the natural compound ascorbic acid (also referred to as vitamin C) has antiproliferative and proapoptotic activities [241], which have also been observed for other antioxidants [242, 243]. Studies conducted by Jin et al. [244] showed that compound Kushen injection (CKI) decreased ROS levels, inhibited proliferation, and promoted apoptosis in AML cells. They also found that the expression of PRX I and PRX II was upregulated, while that of Trx1 was downregulated upon CKI administration. Meanwhile, the hematological parameters of patients with low- to intermediate-risk MDS can be improved by amifostine [245, 246]. Notably, antioxidants combined with specific chemotherapeutic agents result in positive benefits and improved patient survival. Previous research has illustrated improved complete remission and prolonged overall survival in patients with AML when vitamin C was administered in combination with DCA [247]. Interestingly, GSH, vitamins, and N-acetylcysteine appear to be the most common dietary antioxidants used in cancer treatment when combined with chemotherapy/radiotherapy [232, 248].

Collectively, oxidative stress caused by chemotherapy/radiotherapy not only leads to malignant tumor apoptosis but also augments genomic instability, which in turn accelerates disease progression. In particular, MDS and AML are associated with the escalation of oxidative stress [97, 249]. Therefore, an antioxidant approach may be conducive to relaxing DNA impairment and slowing disease progression to a certain extent, and complementary effects may exist between chemotherapy and antioxidants.

### Other therapeutic approaches for MDS

**Fig. 8:**
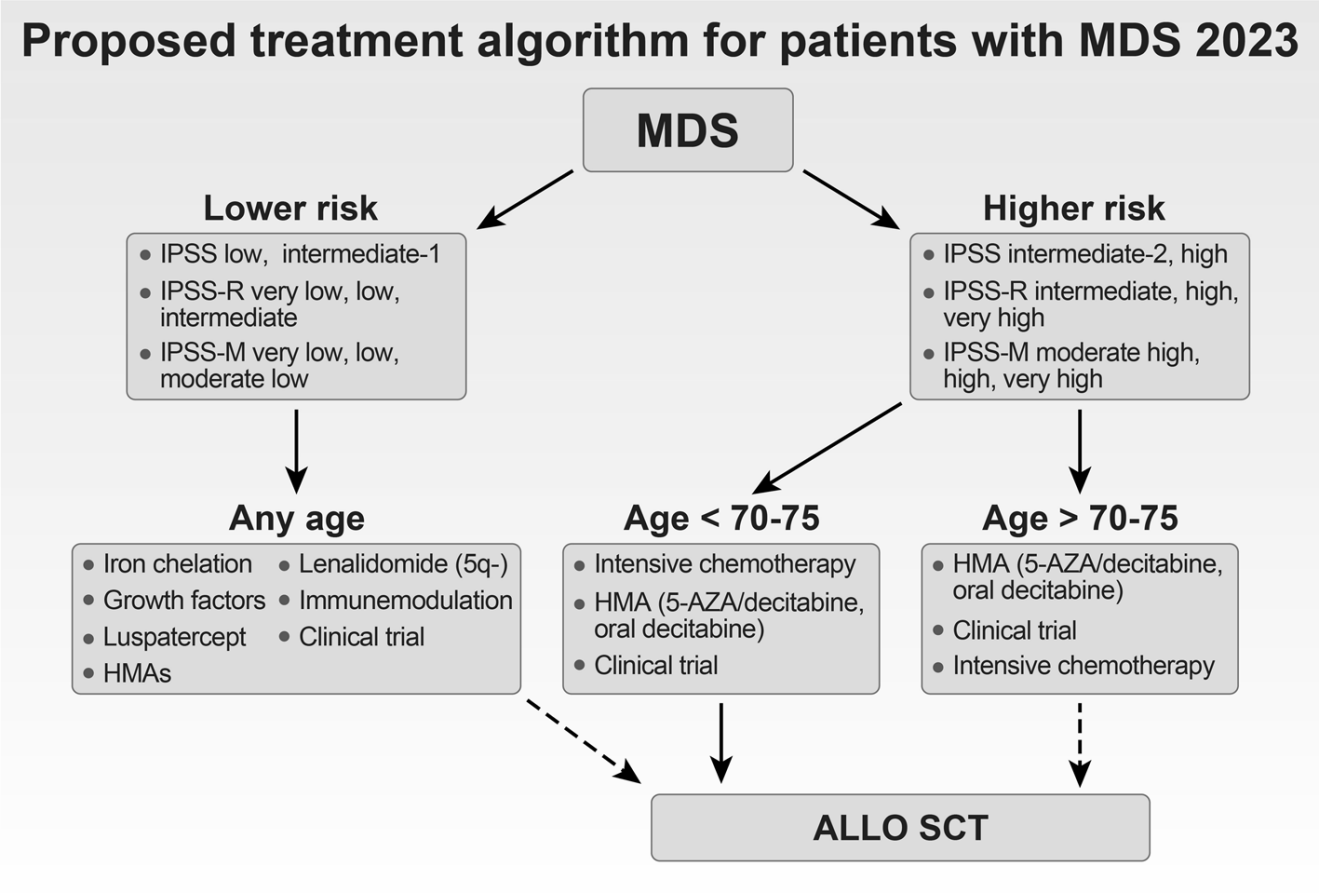
Proposed therapeutic algorithm for patients with MDS

With the heterogeneous nature of MDS comes a need for complex and personalized treatment strategies, and the current treatment therapeutic approaches are based on risk-adapted therapy (by IPSS-R) (Fig. 8). Treatment for patients with LR-MDS (IPSS-R score ≤ 3.5) aims to decrease transfusion requirements, improve living quality and survival, and prevent AML transformation. In the case of patients with HR-MDS, therapy aims to prolong survival.

#### Treatment options for LR-MDS patients

There are several agents for treating LR-MDS patients, including erythropoiesis-stimulating agents (ESAs), immunosuppressive agents, lenalidomide, hypomethylating agents (HMAs), luspatercept, azanucleosides, imetelstat, thrombomimetic agents, canakinumab, as well as allogeneic stem cell transplantation (AlloSCT).

Treatment with ESAs is common practice in patients with anemia and LR-MDS. Results from Platzbecker and colleagues showed a notable increase in erythropoiesis responses and a decrease in transfusion incidence in weeks 5–24 of darbepoetin alfa treatment compared with placebo in patients with LR-MDS, without differences among the groups in terms of the occurrence of thromboembolic events, and transformation to AML [250]. Notably, for eligible MDS patients with or having lost response to ESA, adding the granulocyte colony-stimulating factor can improve response rates [251]. A recognized characteristic of MDS is immune dysregulation, which results in ineffective hematopoiesis and accelerates disease progress [252]. Immune-modulating agent therapy may be therapeutically beneficial for patients with immune dysregulation. An immense amount of clinical trials have delved into immunosuppressive therapy using anti-thymocyte globulin alone or in combination with cyclosporine, displaying trilineage response rates between 16% and 67% [253], and immunosuppressive therapy with alemtuzumab (anti-CD52 antibody) exhibits significant activity and a high response rate in MDS patients [254]. Canakinumab, an interleukin 1 beta (IL-1β) inhibitor, has been explored in patients with LR-MDS. An ex vivo study revealed that the IL-1β-neutralizing antibody canakinumab markedly enhanced the colony-forming activity of HSPCs when cocultured with BM monocytes from SF3B1-mutated LR-MDS [255]. Results from phase II clinical trials confirmed that canakinumab is safe and effectively targets IL-1β signaling, and yielded durable response in LR-MDS patients with single somatic driver mutation in TET2 or DNMT3A [256, 257]. Meanwhile, a multi-institution, open-label, phase 1b/2 clinical trial (NCT04798339) is being carried out to evaluate the toxicity and efficacy of canakinumab in combination with darbepoetin alfa in patients with LR-MDS who have failed prior treatment with an ESA; results are expected in 2024.

Although conventional or reduced-dose HMAs exhibit some activity in patients with LR-MDS, the limited activity and transient response of HMAs following the failure of ESAs as first-line therapy means they are seldom used [258]. Data show that CC-486 (an oral form of azacitidine) has a significant impact on RBC transfusion requirements and platelet responses, yet CC-486 treatment did not improve overall survival [259, 260]. Importantly, there is currently no approval for the use of CC-486 in MDS, and oral HMAs may play a part in patients with LR-MDS in the future. Lenalidomide is considered the recommended therapy for patients with LR-MDS, anemia, good platelet count, and isolated del(5q). Results from a phase III study showed that nearly a third of lenalidomide-treated patients achieve RBC transfusion independence at greater than or equal to 8 weeks, with 8.2 months of median response duration in LR-MDS patients with non-del(5q) who are RBC transfusion dependent and ineligible for or refractory to ESAs [261]. Lenalidomide is capable of elevating the erythroid response rate when combined with ESA in LR-MDS patients with ESA resistance [262]. Notably, in patients with TP53-mutated del(5q) MDS, the response rate to lenalidomide is negatively impacted by TP53 mutation [263, 264].

Luspatercept was approved for patients with LR-MDS by the US FDA in 2020; it can regulate the TGF-beta signaling to ameliorate erythropoiesis and promote late-stage erythroid maturation, and exhibits protracted clinical efficacy [265, 266]. Recently, the COMMANDS trial, aimed at a comparative analysis of the effectiveness and safety of luspatercept and epoetin alfa in managing patients with LR-MDS, showed that luspatercept outperformed epoetin alfa in improving hemoglobin levels and attaining RBC transfusion independence (TI) in ESA-naïve patients with LR-MDS [267]. Nevertheless, these results require long-term follow-up and additional data to confirm. Several studies have evaluated the safety and effectiveness of thrombopoietin agonists for treating patients with LR-MDS. Data from these studies show an impressive rise in platelet responses and lower bleeding event episodes in eltrombopag (thrombopoietin agonist) treated patients when compared with the placebo group, but without significant difference in terms of leukemic transformation [268]. However, more data are needed to support these results. The telomerase inhibitor imetelstat also shows clinical efficacy for patients with LR-MDS. Clinical trials (NCT02598661) observed a significantly durable TI rate in transfusion-dependent patients with LR-MDS after imetelstat treatment, and patients with heavy transfusion and ineligible for or refractory to ESAs can also achieve durable TI and clinical benefit [269, 270].

AlloSCT is currently the only potentially curative therapy for patients with MDS [271]. AlloSCT is not recommended for patients with less advanced disease because a good prognosis is achievable with standard care alone, and the potential favorable survival impact of AlloSCT cannot outweigh the early expected high mortality risk [272]. Patients who received multiple treatments (e.g., lenalidomide, HMAs, luspatercept, azanucleosides, imetelstat, etc.) should be considered for transplantation and clinical trials.

#### Treatment options for HR-MDS patients

Treatment options for patients with HR-MDS are relatively scarce (Fig. 8), and for the bulk of patients for whom intensive chemotherapy is not appropriate, azanucleosides (AZA and DAC) remain the most commonly prescribed medication. Although DAC is approved for MDS treatment in the USA, patients do not benefit from it in terms of survival based on clinical data, and the optimal dosage and treatment schedule of DAC remain uncertain [273–275]. Oral DAC/cedazuridine treatment has proven to be a safe and effective substitute for intravenous DAC for patients with MDS, as shown in a phase III clinical trial (NCT03306264) [276]. AZA has been studied in patients with HR-MDS. The registration trial (AZA-001) found that patients who received azacitidine showed a notable improvement in survival time compared with those who received standard of care, including intensive chemotherapy (24.5 months compared with 15 months) [277]. The progression of MDS to AML transformation was notably delayed, and the need for RBC transfusions and infection rates were also considerably ameliorated. As mentioned, oral azacitidine (CC-486) significantly affects platelet responses and the need for RBC transfusions, and treatment with CC-486 did not increase overall survival. CC-486 was proposed for maintenance therapy after AlloSCT in patients with HR-MDS [278]. In addition, AlloSCT therapy has been discussed above and will not be revisited in this subsection.

## Conclusions and future perspectives

Despite advancements made in the field of medicine, MDS remains an intractable problem that imposes a high disease burden on patients. The heterogeneous nature of MDS necessitates sophisticated and personalized therapeutic strategies, and allogeneic hematopoietic stem cell transplantation remains the only potentially curative therapy for MDS among various approaches [236]. Therefore, the identification of novel therapeutic targets is of paramount importance.

ROS have been implicated in metabolic regulation, stress responses, and redox signaling. As ROS accumulation and oxidative damage are strongly associated with various pathologies, including MDS and several forms of myeloid leukemia, interest in ROS research has continued to grow in recent years. The observation of increased ROS and OS in MDS, especially in patients with LR-MDS, suggests that ROS may be an attractive therapeutic target and that ROS modulation therapy could be a useful approach for MDS treatment. Indeed, the prooxidant approach is the preferred choice for clinical first-line treatment because chemotherapy triggers malignant tumor regression and apoptosis by elevating ROS levels and OS. Furthermore, antioxidant approaches can augment the cytotoxicity of chemotherapy and protect nonmalignant cells from oxidative damage. Finally, identifying the source and species of ROS produced by MDS and targeting control-specific ROS-mediated signaling pathways by designing redox drugs may be viable strategies for the management of MDS in the future. This review highlights ROS production, which may play a pivotal role in the pathogenesis and treatment response of MDS.

## Data Availability

Not applicable.

## Abbreviations

^1^O_2_: Singlet oxygen
ABT-199: Venetoclax
ALA: Alpha lipoic acid
AlloSCT: Allogeneic stem cell transplantation
AML: Acute myeloid leukemia
AOs: Amine oxidases
APL: Acute promyelocytic leukemia
ASXL1: ASXL transcriptional regulator 1
Atg7: Autophagy related 7
ATM: Ataxia-telangiectasia mutated
ATO: Arsenic trioxide
ATRA: All-*trans* retinoic acid
AZA: Azacitidine
Bax: B‑cell lymphoma 2 associated X protein
BCL-2: B‑cell lymphoma 2
BID: BH3 interacting domain death agonist
BM: Bone marrow
BMNCs: Bone marrow nucleated cells
CAR: CXCL12-abundant reticular
CAT: Catalase
CDC 39: CCR4-NOT transcription complex subunit 1
CKI: Compound Kushen injection
CML: Chronic myeloid leukemia
CoQ10: Coenzyme Q10
COX2: Mitochondrially encoded cytochrome C oxidase II
CSNK1A1: Casein kinase 1 alpha 1
CXCL12: CXC-chemokine ligand 12
CXCL4: CXC chemokine ligand 4
CXCL4: CXC-chemokine ligand 4
CXCR4: C-X-C motif chemokine receptor 4
CYP: Cytochrome P450
CYP2D6: Cytochrome P450 2D6
CYP2E1: Cytochrome P450 2E1
CYP3A4: Cytochrome P450 3A4
CYP450: Cytochrome P450
CYP4A11: Cytochrome P450 4A11
Cyto: Cytosol
DAC: Decitabine
DARC: Duffy antigen receptor for chemokines
DFX: Deferasirox
DNMT3A: DNA methyltransferase 3 alpha
d-ROMs: Dacron-reactive oxygen metabolites
DRP1: Dynamin-related protein 1
DUOX1/2: Dual oxidase 1/2
EF: Elongation factor
ER: Endoplasmic reticulum
ERFE: Erythroferrone
ES: Endosome
ESA: Erythropoiesis-stimulating agents
ETC: Electron transport chain
ExC: Extracellular space
FA: Fanconi anemia
FGF1: Fibroblast growth factor 1
FLT3-ITD: Fms-like receptor tyrosine kinase 3-internal tandem duplication
FOXO: Forkhead box O
gp130: Glycoprotein 130
GPX: Glutathione peroxidase
GSH: Glutathione
GSSG: Oxidized glutathione
H_2_O_2_: Hydrogen peroxide
HIF1: Hypoxia-inducible factor 1
HMA: Hypomethylating agents
HO-1: Heme oxygenase 1
HOBr: Hypobromous acid
HOCl: Hypochlorous acid
HPSCs: Human pluripotent stem cells.
HR-MDS: High-risk myelodysplastic syndromes
HSCs: Hematopoietic stem cells
HSPCs: Hematopoietic stem and progenitor cells
ICT: Iron chelation therapy
IDH1/IDH2: Isocitrate dehydrogenase 1/2
IL-1β: Interleukin 1 beta
IL-7/10: Interleukin-7/10
IOL: Iron overload
IPSS-R: Revised international prognostic scoring system
LEPR: Leptin receptor
LR-MDS: Low-risk myelodysplastic syndromes
MDS: Myelodysplastic syndromes
Mfn2: Mitofusin
MMP: Mitochondrial membrane potential
MPO: Myeloperoxidase
mROS: Mitochondrial ROS
MSCs: Mesenchymal stromal cells
Mt ETC: Mitochondrial electron transport chain
MTCH2: Mitochondrial carrier homologue 2
mTOR: Mammalian target of rapamycin
MYH11: Myosin heavy chain 11
NAC: N-acetylcysteine
NAD^+^: Nicotinamide adenine dinucleotide
NADPH: Nicotinamide adenine dinucleotide phosphate
Nes: Nestin
NFAT: Nuclear factor of activated T cells
NF-κB: Nuclear factor κB
NG2: Neural-glial antigen 2
NHD13: NUP98-HOXD13
NLRP3: NLR family pyrin domain containing 3
NO^⋅^: Nitric oxide
NOX: NADPH oxidase
NOX1-5: NADPH oxidase 1–5
NOXA1: NADPH oxidase activator 1
NOXO1: NADPH oxidase organizer 1
Nrf2: Nuclear factor erythroid-2 related factor 2
O_2_: Oxygen
O_2_^⋅−^: Superoxide anion radicals
O_3_: Ozone.
OH^⋅^: Hydroxyl radicals
ONOO^−^: Peroxynitrite anion
OPN: Osteopontin
OS: Oxidative stress
OSM: Oncostatin M
OXPHOS: Oxidative phosphorylation
PGE2: Prostaglandin E2
PM: Plasma membrane
PRX: Peroxiredoxin
R^⋅^: Hydrocarbon radicals
RARα: Retinoic acid receptor alpha
RBC: Red blood cell
RH: Hydrocarbons
RIPK1: Receptor interacting serine/threonine kinase 1
RNS: Reactive nitrogen species
RO^⋅^: Alkoxyl radicals
ROO^⋅^: Peroxyl radicals
ROOH: Organic hydroperoxides
ROS: Reactive oxygen species
RPS14: Ribosomal protein S14
RUNX1: RUNX family transcription factor 1
SCF: Stem cell factor
SF3B1: Splicing factor 3b subunit 1
SNS: Sympathetic nervous system
SOD: Superoxide dismutase
SRSF2: Serine and arginine rich splicing factor 2
TCA: Tricarboxylic acid
T-dCyd: Thio-deoxycytidine
TET2: Tet methylcytosine dioxygenase 2
TGFβ: Transforming growth factor-β
TI: Transfusion independence
TNF: Tumor necrosis factor
T_reg_ cells: Regulatory T cells
Trx: Thioredoxin
TSC: Tuberous sclerosis complex
U2AF1: U2 small nuclear RNA auxiliary factor 1
VCAM1: Vascular cell adhesion molecule 1
Vwf: Von Willebrand factor
XO: Xanthine oxidase
XOR: Xanthine oxidoreductase
